# Proposal for standardized ultrasound analysis of the salivary glands: Part 1 submandibular gland

**DOI:** 10.1002/lio2.1224

**Published:** 2024-02-14

**Authors:** Henry T. Hoffman, Michael Koch, Robert Lee Witt, William R. Ryan, Johannes Zenk, Philippe Katz, Rahmatullah Rahmati, Christopher Rassekh, Francisco Donato, Timothy M. McCulloch, Arjun S. Joshi, Jolie Lien Chang, M. Boyd Gillespie, Priscilla F. A. Pichardo, Lisa Ann Orloff, Antoino Marcelino, Piper Wenzel, David Cohen, Christopher E. Fundakowski, David M. Cognetti, Rohan R. Walvekar, Antonio Bertelli, Harry Quon, Carryn Anderson, Bruno Policeni, Gordy Siegel

**Affiliations:** ^1^ University of Iowa Iowa City Iowa USA; ^2^ University of Erlangen‐Nuremberg Erlangen Germany; ^3^ Christiana Care/Thomas Jefferson University Philadelphia Pennsylvania USA; ^4^ University of California San Francisco San Francisco California USA; ^5^ University Hospital Augsburg Augsburg Germany; ^6^ Institut d'Explorations Fonctionnelles des Glandes Salivaires Paris France; ^7^ Harvard Medical School Boston Massachusetts USA; ^8^ University of Pennsylvania Perelman School of Medicine Philadelphia Pennsylvania USA; ^9^ University of Iowa Hospitals and Clinics Iowa City Iowa USA; ^10^ University of Wisconsin School of Medicine and Public Health Madison Wisconsin USA; ^11^ The George Washington University Washington District of Columbia USA; ^12^ University of California San Francisco California USA; ^13^ University of Tennessee Health Science Center College of Medicine Memphis Tennessee USA; ^14^ Geisinger Health System Danville Pennsylvania USA; ^15^ Stanford University Stanford California USA; ^16^ Kaiser Permanente Los Angeles Medical Center Los Angeles California USA; ^17^ Thomas Jefferson University Hospital Philadelphia Pennsylvania USA; ^18^ Louisiana State University HSC New Orleans Louisiana USA; ^19^ Faculdade de Ciencias Medicas da Santa Casa de Sao Paulo Brazil Sao Paulo Brazil; ^20^ Johns Hopkins Medical Institutions Campus Baltimore Maryland USA; ^21^ Northwestern University Feinberg School of Medicine Chicago Illinois USA

**Keywords:** anatomic subsites, color doppler, salivary glands, shear wave elastography, submandibular, ultrasound

## Abstract

**Objectives:**

The Salivary Gland Committee of the American Academy of Otolaryngology‐Head and Neck Surgery seeks to standardize terminology and technique for ultrasonograpy used in the evaluation and treatment of salivary gland disorders.

**Methods:**

Development of expert opinion obtained through interaction with international practitioners representing multiple specialties. This committee work includes a comprehensive literature review with presentation of case examples to propose a standardized protocol for the language used in ultrasound salivary gland assessment.

**Results:**

A multiple segment proposal is initiated with this focus on the submandibular gland. We provide a concise rationale for recommended descriptive language highlighted by a more extensive supplement that includes an extensive literature review with additional case examples.

**Conclusion:**

Recommendations are provided to improve consistency both in performing and reporting submandibular gland ultrasound.

## Graphical abstract

The AAO‐HNS Salivary Gland Committee in collaboration with international experts proposes a standardized approach to submandibular gland ultrasound analysis focused on technique and nomenclature. Consensus regarding the naming of subsites within the submandibular gland is needed to improve communication about abnormalities detected and to direct the use of more sophisticated assessment schemes such as shear wave analysis targeted to specific regions. Consistency in this terminology should lead to improved reproducibility of findings.

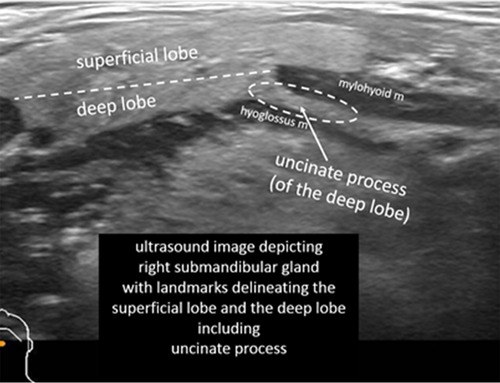

## 1 INTRODUCTION

Point‐of‐care ultrasound (POCUS) analysis was described by Moore and Copel in 2011 as an evaluation performed and interpreted immediately at the bedside by the clinician.^1^ Advances in technology have made ultrasound “*user‐friendly for all practitioner*s” with a recent report addressing POCUS by Liao et al supporting it as “*essential for clinical practice as well as for training in the field of otolaryngology and head and neck surgery*.”^2^

This AAO‐HNS Salivary Gland Committee sponsored proposal to standardize technique and nomenclature initially arose from POCUS as practiced in Otolaryngology. However, the recommendations are designed to be broadly applicable to clarify established protocols in other settings including the consultative comprehensive technician‐performed examinations done in Radiology departments. Multidisciplinary specialists were called on to refine these recommendations to cross boundaries between specialties and support broad acceptance.

This work identifies assessment techniques and defines subsites to improve consistency in performing and reporting ultrasound evaluations. This approach to standardization focuses on static images but is also applicable to terminology used in review of video clips as has been advocated to improve interrater reliability.^3^ The technique of ultrasound video‐imaging review has also been supported by the OMERACT (Outcome Measures for Rheumatoid Arthritis Clinical Trials) group. OMERACT has published a semi‐quantitative approach to provide a global assessment of salivary gland (primarily parotid) abnormalities associated with Sjogren's disease through a review of video‐imaging to characterize the degree of pathology seen throughout the gland.^4^, ^5^, ^6^ An updated report by Tang et al supported the value of applying this OMERACT scoring system to the analysis of static images of the salivary glands.^7^ However, their report offered only a broad review of “*typical static grayscale images*” in their report without identifying which subsites within the glands were analyzed.

Radiomics is defined as a method to extract information from medical images beyond that provided by visual inspection and has received intense scrutiny with MRI and CT imaging. Ultrasound radiomics is a developing field with acknowledged limitations due to inconsistency in image acquisition and difficulties in calibrating quantitative methods.^8^ Subjective grading of salivary ultrasound images through semi‐quantitative classification schemes has been supplemented by quantitative assessment employing shear wave elastography to refine analysis.^4^, ^6^, ^9^, ^10^, ^11^ Assessment by many different methods of ultrasound analysis generally lack consistency in identifying specific anatomic regions (subsites) within the salivary glands and rarely report differences between regions in the same gland.^12^, ^13^

Shear wave elastography is a quantitative ultrasound method used to determine the velocity of tissue displacement resulting from a secondary “push pulse” produced by the ultrasound probe. The speed of tissue displacement (shear wave) correlates with the tissue stiffness or fibrosis from the selected “regions of interest” evaluated.^14^ Standardization to identify specific subsites within the salivary glands is needed to provide consistency for many reasons, including the value in the identification of “regions of interest” needed for shear wave measurements.^15^, ^16^, ^17^, ^18^

This report addresses the standardization of salivary gland ultrasound nomenclature and measurement technique with an additional focus on the reporting to include specific subsites within the gland. Recommendations are designed to provide logical terminology and consistency in salivary gland imaging intended for clinical and research applications within the evolving field of ultrasound radiomics.^19^

The following recommendations derive both from expert opinion of the contributing authors and an extensive literature review that is detailed in the accompanying supplement along with representative case examples.

## 2 RECOMMENDATIONS

### 2.1 Submandibular gland anatomy and subsites

We propose that the submandibular gland is comprised a superficial lobe and a deep lobe. The deep lobe also includes an anterior projection termed the uncinate process partially surrounding Wharton's duct. The traditional use of the term ‘lobe’ is clearly defined when applied to the lung to identify segments determined by bronchial branching.^20^ The pulmonary lobes are also identifiable through the classic definition that “a lobe is part of an organ defined by a fissure seen at the surface of the organ”.^21^

The superficial ‘lobe’ of the submandibular gland is more accurately termed the superficial ‘aspect’ or ‘portion’. Discrimination from the deep ‘aspect’ of the gland is determined by its relationship to an external structure (the mylohyoid muscle) and not an internal organization or an identifiable fissure. Similarly, the liver has been broadly classified as having the right and left lobes that do not correspond to the more critical subdivision into multiple sectors or segments discriminated by blood supply and biliary flow.^22^ We acknowledge the more accurate division of the submandibular gland into two “aspects”—the superficial and deep portions. However, due to established conventions, the more widely used terminology employing the term ‘lobes’ is also reasonable.

Also considered within the anatomic unit of the submandibular gland are the surrounding capsule as well as internal vasculature where surrounded by gland parenchyma (Table 1).^23^ The capsule of the submandibular gland has been identified as a continuation of the investing layer of the deep cervical fascia.^24^ As per O'Daniel, this fascia is thicker anteriorly (superficial aspect) and thinner posteriorly (deep aspect).^25^ A normal capsule to the submandibular gland will be identified with a hyperechoic appearance. The loss of definition to the gland border is considered a sufficiently abnormal finding that its absence is used in ultrasound grading scales to support the diagnosis of Sjogren's syndrome.^10^, ^13^, ^26^ Obesity, diabetes and sialosis have also been identified to be associated with an invisible deep (posterior) border.^3^, ^27^

**TABLE 1:** Proposed terminology to define subsites and regions within the submandibular gland.

Ultrasound Anatomic Definitions		
	Entire Submandibular Gland	Includes uncinate process, Warton's duct, gland capsule and blood vessels within the gland parenchyma
	Lobes (Aspects)	
Superficial lobe (aspect)	Superficial to mylohyoid including the aspect of gland posterior to the mylohyoid	Includes branches of facial artery and vein encompassed by parenchyma
Deep lobe (aspect)	Deep to mylohyoid includes uncinate process as well as a portion of gland deep to the line of mylohyoid along the full extent of the gland	Includes branches of the facial artery and vein encompassed by parenchyma
	Subunits	
Uncinate process	Anterior extension of the deep lobe of the submandibular gland between the mylohyoid and hyoglossus muscles and below intraoral mucosa	
Posterior and Superior vascularized gland	Includes branches of the facial blood vessels (artery and vein) within the profile of the gland parenchyma	
Gland capsule (border)	Capsule is anatomically thinner on deep aspect of gland	Ultrasound appearance of capsule (border) is diminished by processes including obesity, sialosis, irradiation and autoimmune sialadenitis

**FIGURE 1:**
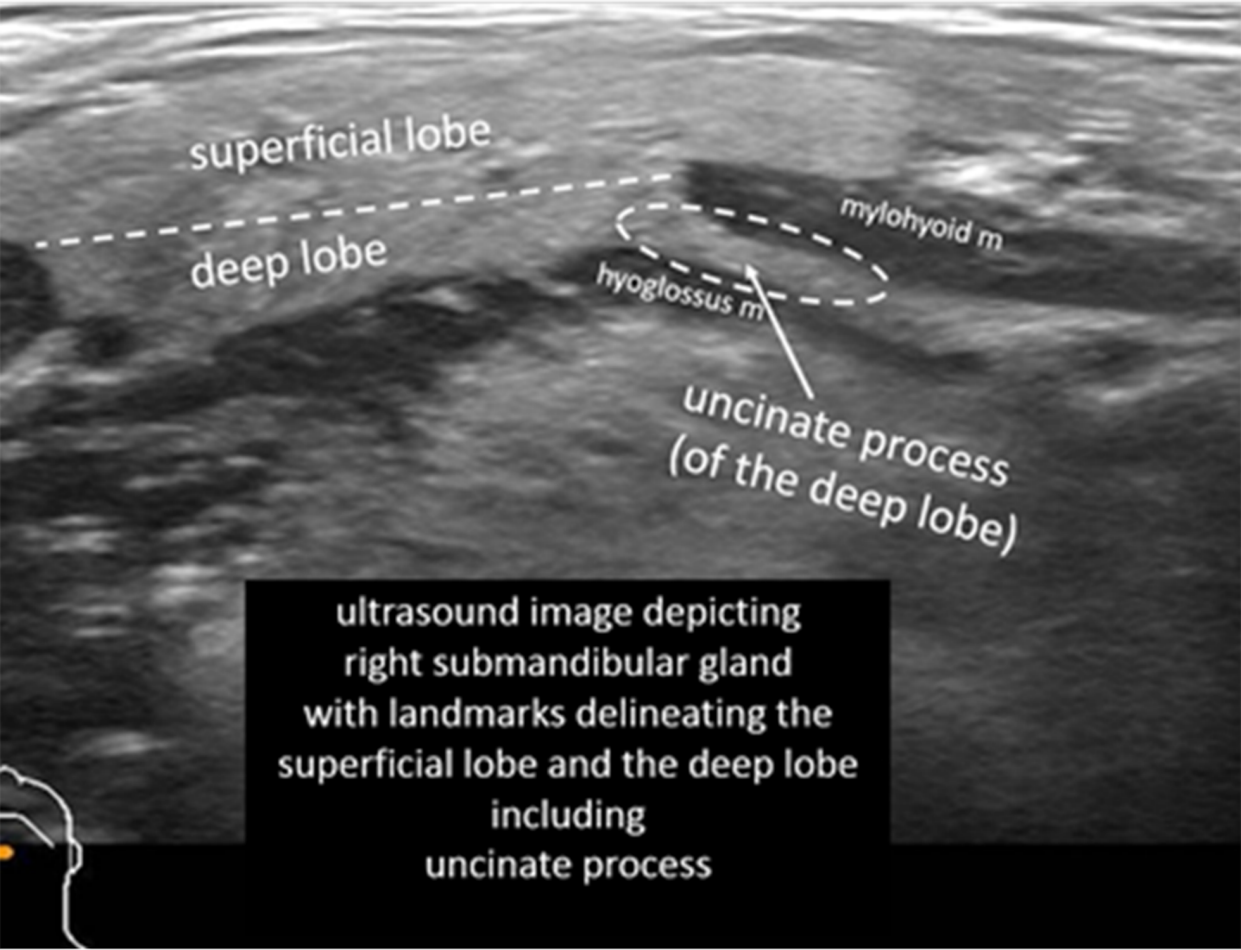
Ultrasound (14–5 MHz linear probe transverse) identifying superficial and deep lobes and the uncinate process of the left submandibular gland (with approval from Hoffman HT (ed) Iowa Head and Neck Protocols <Submandibular Gland Anatomy: The Uncinate Process of the Deep Lobe|Iowa Head and Neck Protocols (uiowa.edu) > accessed April 2, 2023.

The superficial lobe is distinguished from the deep lobe (including the uncinate process) by a line running parallel to the transverse axis of the gland as determined by the posterior margin of the mylohyoid muscle (Figure 1).

**FIGURE 2:**
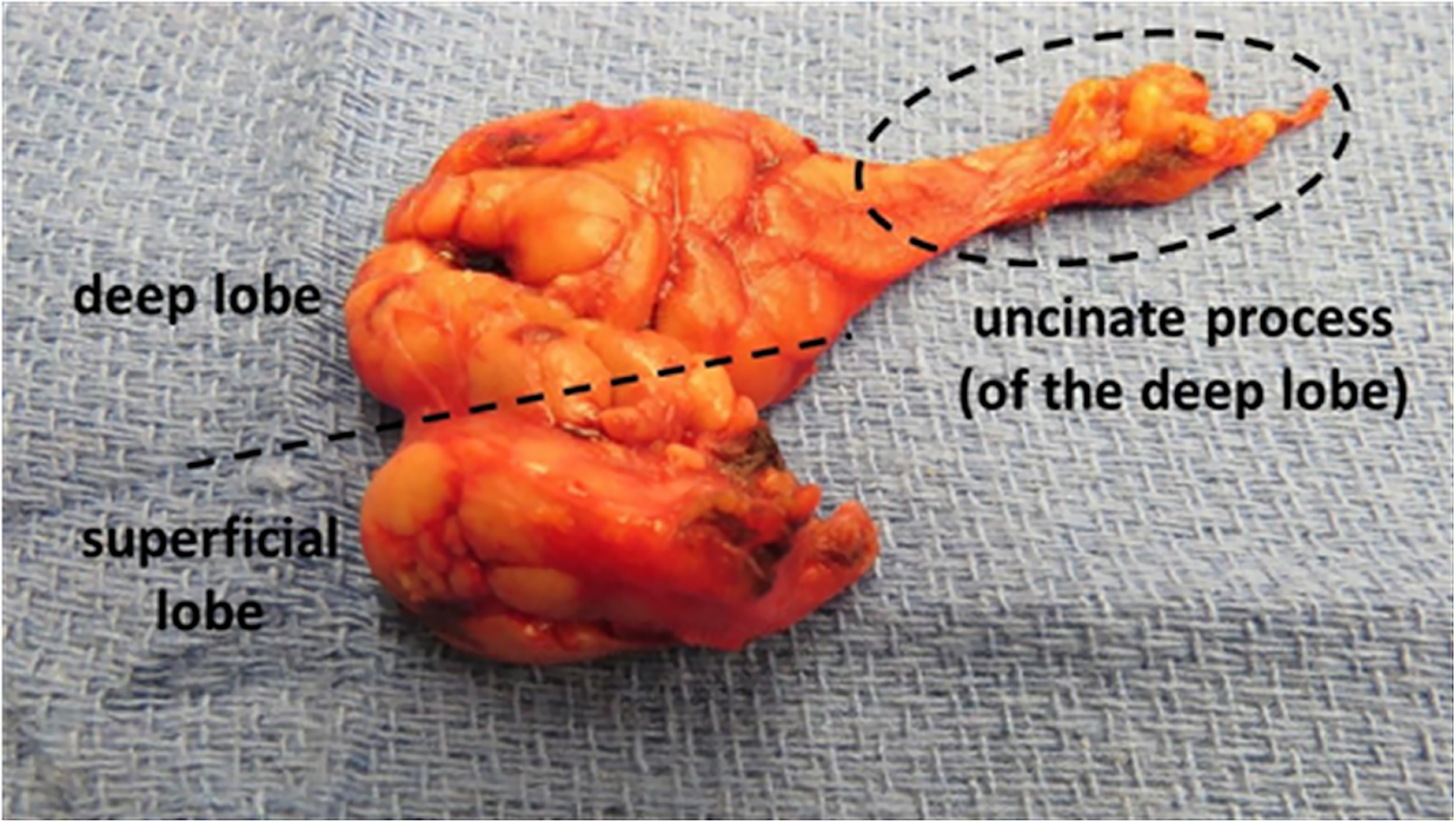
Specimen of a submandibular gland resection extended to include the uncinate process and duct after previous total sublingual gland resection for ranula. The pathological review showed no remaining sublingual tissue. (with approval from Hoffman HT (ed) Iowa Head and Neck Protocols <Submandibular Gland Anatomy: The Uncinate Process of the Deep Lobe|Iowa Head and Neck Protocols (uiowa.edu) ≥accessed April 2, 2023.

The deep lobe includes the uncinate process, which is defined as the anterior extension of the submandibular gland between the mylohyoid and hyoglossus muscles (Figure 2).

**FIGURE 3:**
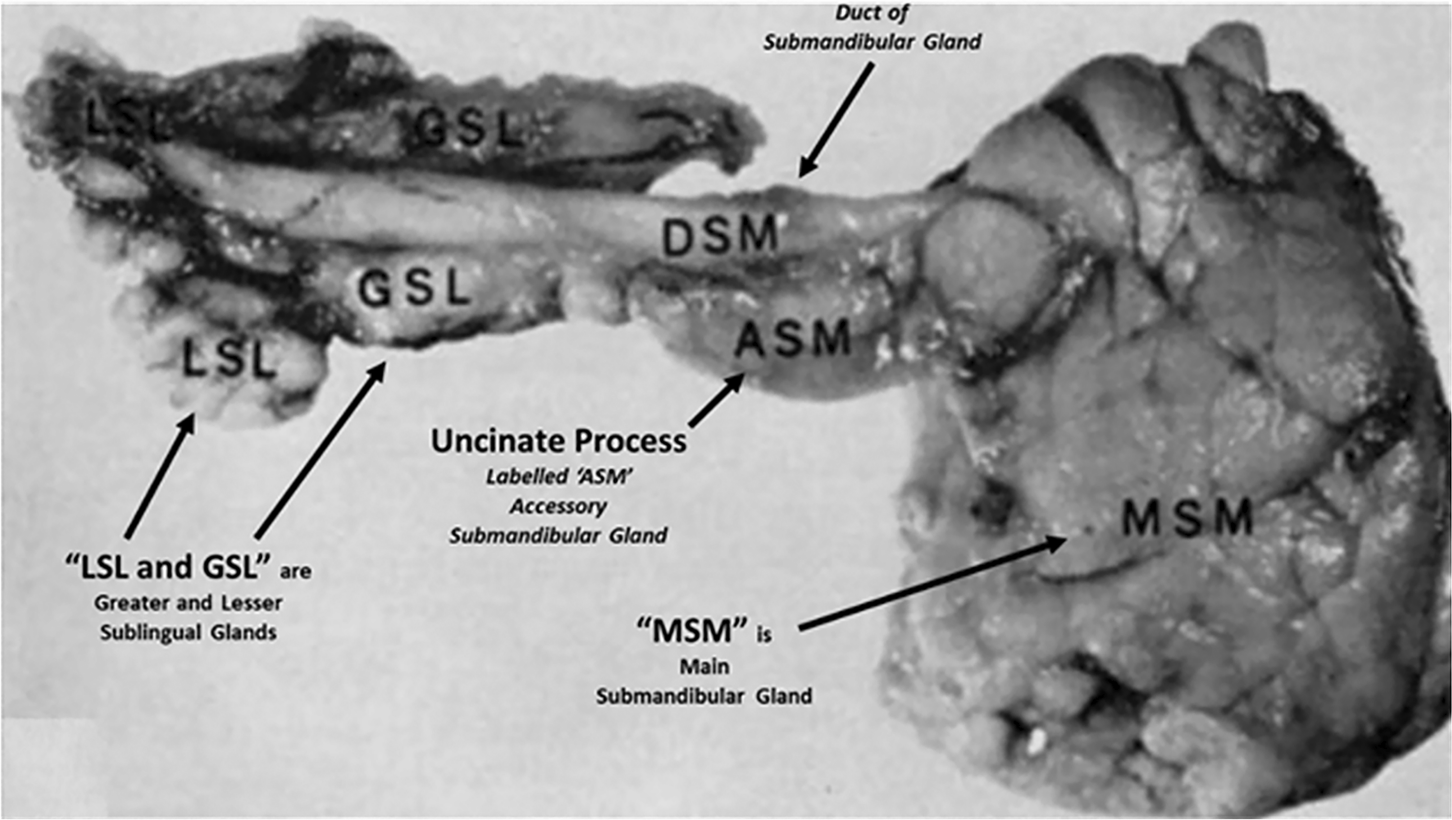
Medial (deep) view of right submandibular complex with relabeling of the submandibular duct (DSM) associated with the uncinated process (ASM, “accessory submandibular gland”) in relation to sublingual glands (GSL, “greater sublingual gland,” LSL, “lesser sublingual gland”) (with permission Leppi 1967).

This uncinate process of the submandibular gland includes parenchymal tissue surrounding Wharton's duct and may be difficult to discriminate on ultrasound examination from sublingual gland tissue. Leppi performed elegant cadaveric dissections to discriminate the uncinate process from the sublingual gland and identified “variable groupings” of submandibular tissue above the mylohyoid muscle (Figure 3).^28^ Although he termed these anterior extensions of the submandibular gland as either “accessory glands” or “secondary glands,” we feel the term “uncinate process” of the submandibular gland most effectively describes this extension.

**FIGURE 4:**
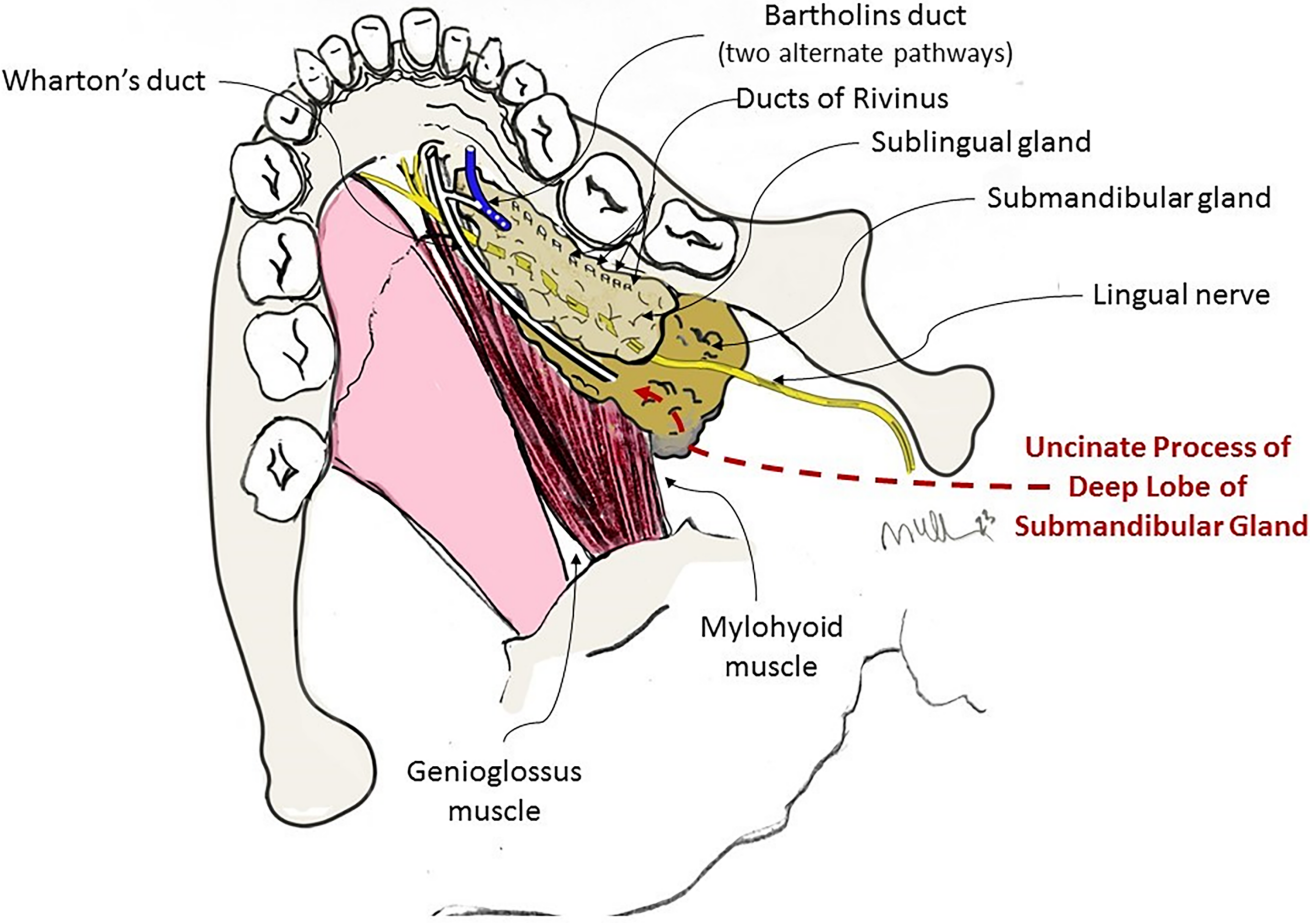
Diagram of the floor of mouth highlighting the variable drainage from the sublingual gland (Bartholin's duct and Ducts of Rivinus) in demonstrating the relationships to the uncinate process of the deep lobe of the submandibular gland. (with approval from Hoffman HT (ed) Iowa Head and Neck Protocols <Submandibular Gland Anatomy: The Uncinate Process of the Deep Lobe|Iowa Head and Neck Protocols (uiowa.edu) > accessed June 18, 2023).

The anatomy of the floor of the mouth is complicated by the variable relationships not only between the sublingual gland and Wharton's duct but also by variety in the extent of the uncinate process (Figure 4).

**FIGURE 5:**
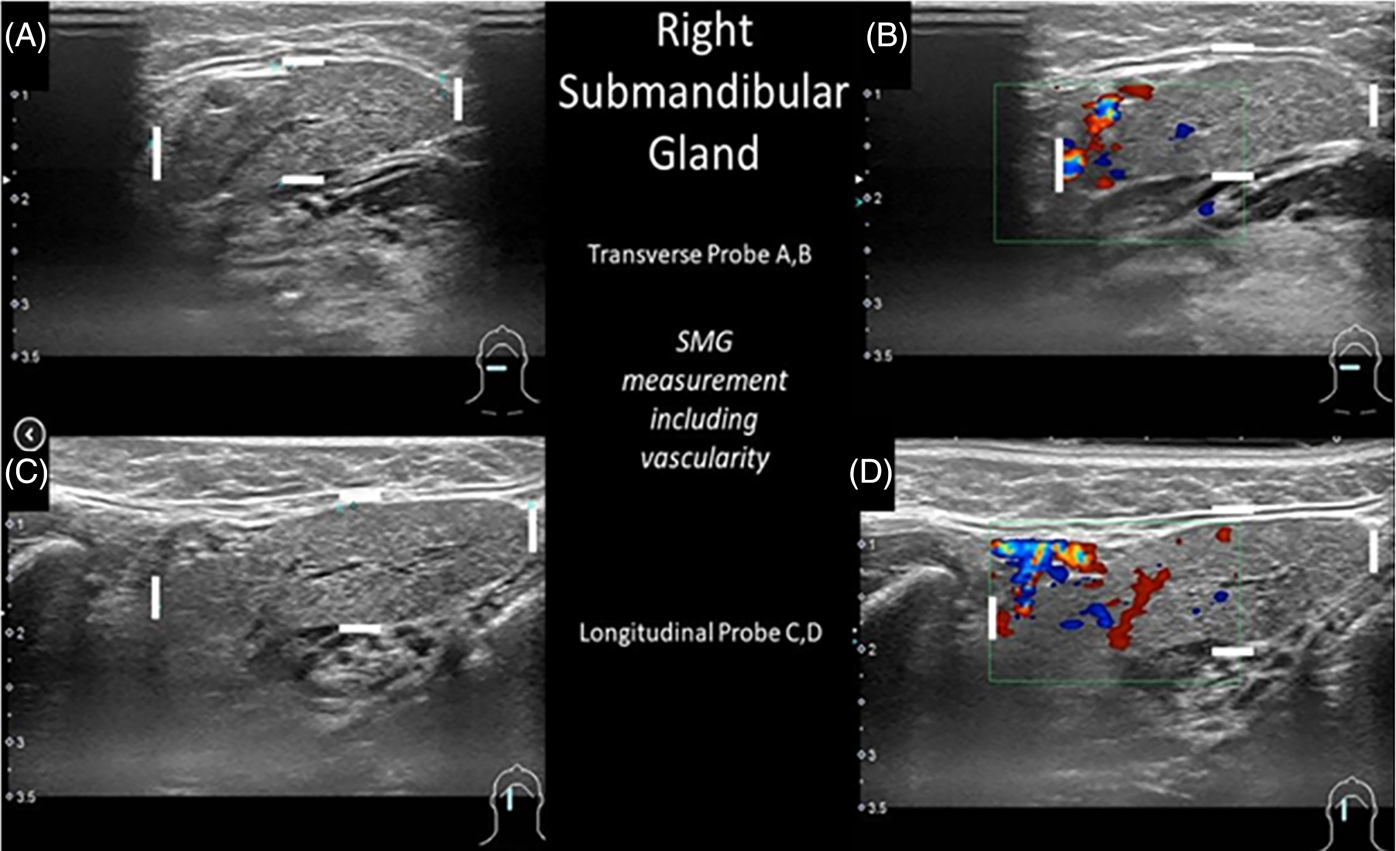
Ultrasound of right submandibular gland assessed with 5–14 MHz linear probe in transverse (3A, B) and longitudinal (3C, D) orientations identifying measurement of the size of the gland to include the blood vessels imaged with color doppler. (with approval from Hoffman HT (ed) Iowa Head and Neck Protocols <Submandibular Gland Anatomy: Vascular Supply—Ultrasound Imaging with Color Doppler https://medicine.uiowa.edu/iowaprotocols/submandibular‐gland‐anatomy‐vascular‐supply‐ultrasoundimaging‐color‐doppler> accessed June 18, 2023).

Anatomic variation to the posterior and superior aspects of the submandibular gland may create difficulty in measuring the extent of parenchyma due to variability in the vascular structures in this region. Ultrasound with color doppler is useful not only in discriminating between blood vessels and non‐vascular ductal elements but may direct more accurate size measurement of the submandibular gland by identifying where blood vessels are surrounded by gland parenchyma (Figure 5).^3^, ^29^

Although the arterial blood supply to the submandibular gland may also arise from the lingual, deep lingual, and external carotid arteries, the dominant blood supply is from the facial artery (including the submental branch of the facial artery). Li et al identified that the facial artery runs along a “*groove*” within the submandibular gland and can be surrounded by the cortex of the gland.^29^, ^30^

### 2.2 Salivary ultrasound assessment techniques

#### 2.2.1 Equipment

As is recommended by the American Institute of Ultrasound in Medicine (AIUM) for “extracranial head and neck ultrasound evaluations”, we support use of a linear transducer for salivary gland evaluation.^31^ The AIUM recommends a mean frequency of 10 to 14 MHz probe and notes that a greater depth of penetration may warrant use of lower frequencies.

#### 2.2.2 Technique

The American Institute of Ultrasound in Medicine (AIUM) has published practice parameters for the documentation of an ultrasound examination.^31^ These generalized guidelines emphasize that “*accurate and complete documentation and communication are essential for high‐quality patient care*.” Recording of anatomic measurements is recommended when appropriate. A separate publication from the AIUM addressing head and neck ultrasound offers more specific recommendations to identify that reporting of “*focal abnormalities within the salivary glands should include the size in 3 dimensions*”.^14^

**FIGURE 6:**
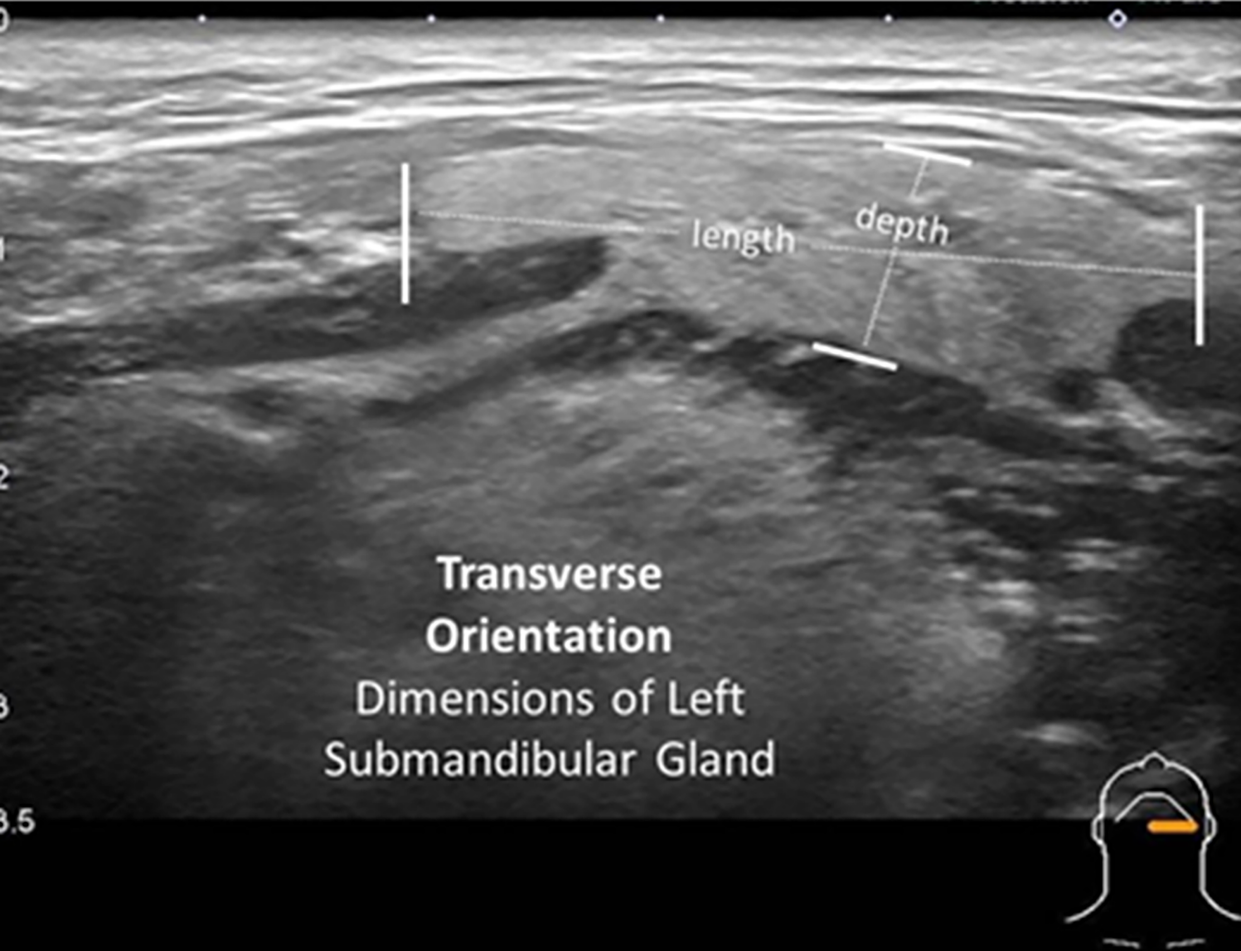
Transverse orientation (with slight obliquity) of the 14–5 MHz linear ultrasound probe identifies measurement of the length and depth of the left submandibular gland.

**FIGURE 7:**
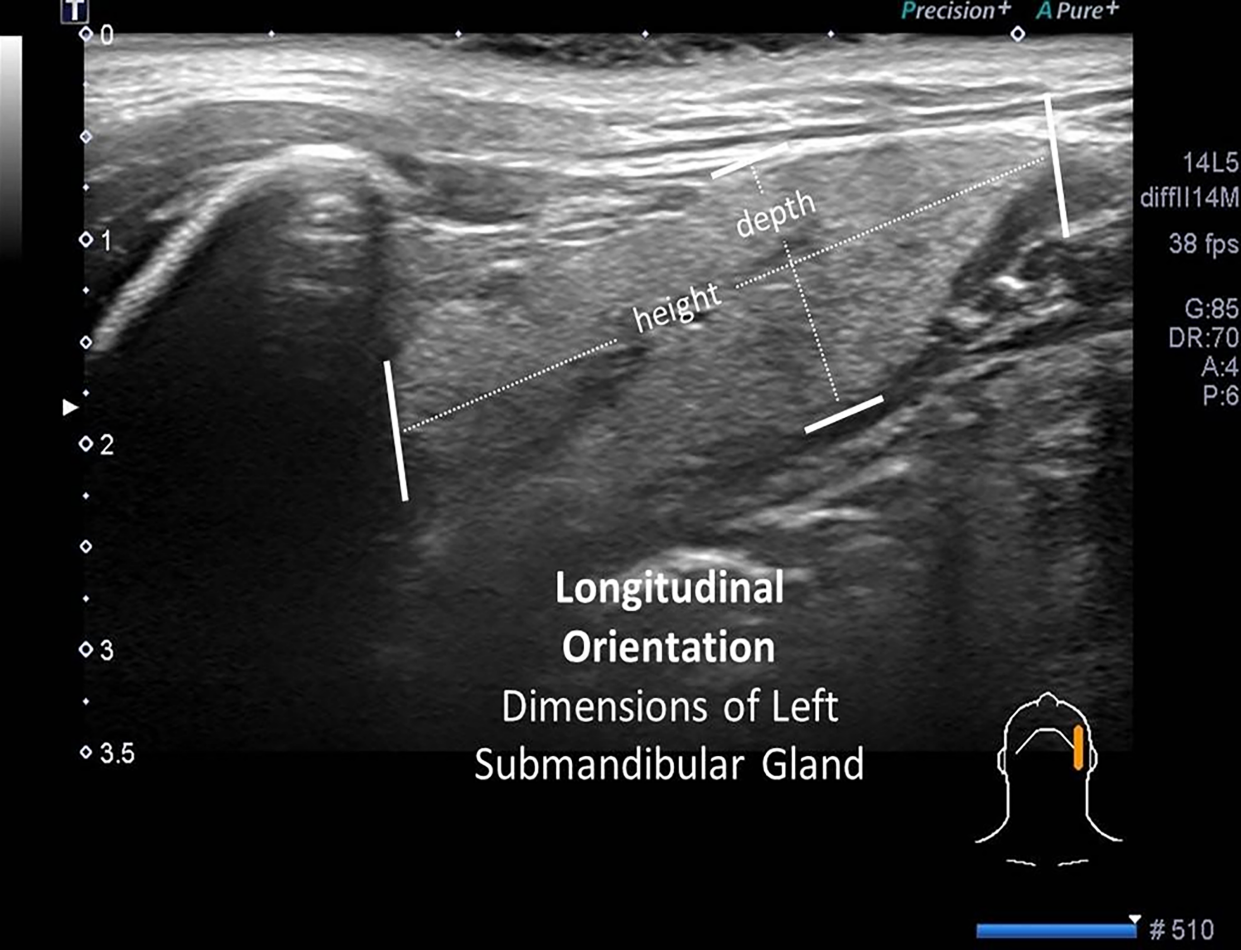
Longitudinal orientation of the 14–5 MHz linear ultrasound probe (with slight obliquity) identifies the height and depth of the left submandibular gland.

We propose terminology to standardize the assessment and reporting of ultrasound probe positioning and assessment (Table 2) highlighted by representative ultrasound images (Figures 6 and 7). Ultrasound assessment of submandibular size usually does not include consideration of the anterior extension of Wharton's duct with surrounding tissue. The anterior extension of Wharton's duct can occasionally be difficult to image due to shielding from the bone of the mandible despite the use of sonopalpation.^32^

**TABLE 2:** Terms addressing probe orientation in reporting SMG ultrasound measurement.

Analysis should be done in 2 planes—anatomic limitations may require oblique planes.			
AAO‐HNS Salivary Gland Committee Recommendation 2023 dimensions recorded are widest for each orientation the uncinate process is not included in reporting dimensions. (images courtesy of Francisco Donato)	**Length (L)** Anterior to posterior **Transverse** **Probe** 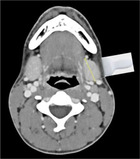 Axial CT with diagram showing **Length** measurement with transverse probe position	**Height (H)** Inferior to superior **Longitudinal** **Probe** 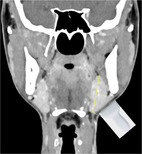 Coronal CT with diagram showing **Height** measurement with longitudinal probe position	**Depth (D)** Lateral to medial. **Transverse** **Probe** **Width (W)** is also a commonly used term but not as precise **Depth (D)** 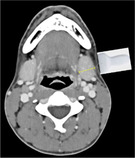 Axial CT with diagram showing **Depth** measurement with transverse probe position

**FIGURE 8:**
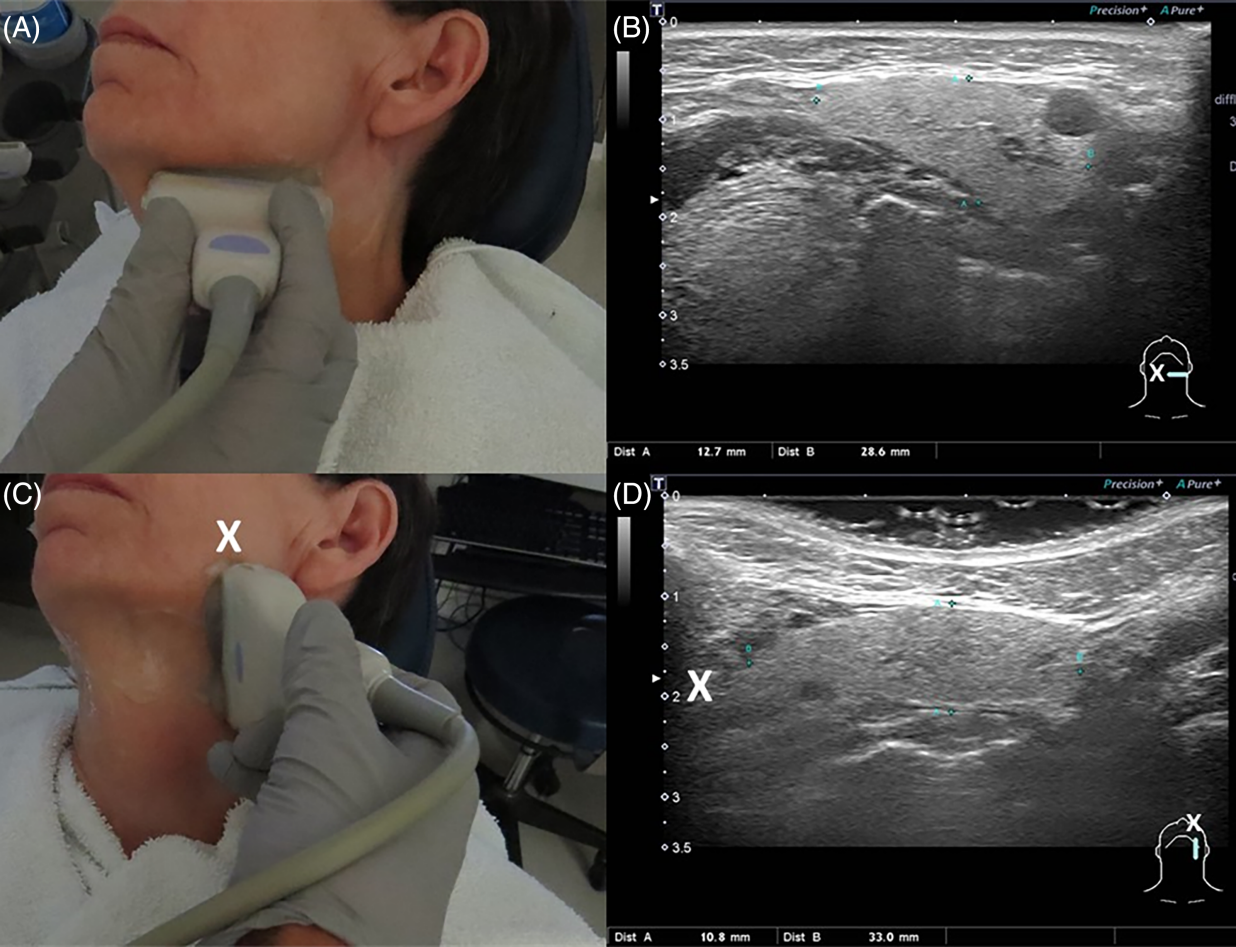
The linear 6.5 cm × 0.75 cm 14–5 megahertz (MHz) probe (Canon Aplio 500 ultrasound unit) is positioned slightly oblique in transverse (parallel to body of mandible A and B) and longitudinal (perpendicular to body of mandible C and D) orientations to image the left submandibular gland. Note the change in orientation delineated by “**X**” showing the anatomically superior aspect of the gland (C) on longitudinal view is portrayed on the left side of the image (D) (with approval from Hoffman HT (ed) Iowa Head and Neck Protocols < Salivary ultrasound standardized diagnostic approach and report https://medicine.uiowa.edu/iowaprotocols/salivary‐ultrasound‐standardized‐diagnostic‐approach‐and https://medicine.uiowa.edu/iowaprotocols/salivary‐ultrasound‐standardized‐diagnostic‐approach‐and‐reportreport> accessed June 18 2023).

Although the terms “transverse” and “longitudinal” may be used without modifiers, some degree of obliquity relative to the central axis is usually introduced to permit examination with the probe parallel (transverse) and perpendicular (longitudinal) to the body of the mandible usually initiated perpendicular to the skin surface (Figure 8).

Additional imaging with the probe position altered from this initial orientation is generally required to identify ductal and hilar stones.^33^ Angling the transversely positioned probe in a rostral direction (under the mandible) is often needed and may be supplemented by intraoral digital depression of the floor of the mouth to deliver structures into the field of view by the process termed “sono‐palpation.”

The terminology addressing probe orientation is confusing and differs based on whether the long axis of the patient or the long axis of the structure studied is emphasized.^34^ The term “transverse” is recommended to describe imaging with the probe positioned parallel to the body of the mandible—similar to “axial” or “cross‐sectional”—in a plane that is perpendicular to the long axis of the patient.^35^, ^36^, ^37^ Differences persist in the literature regarding terminology to description the image of the gland as identified by this transverse probe placement. The word “longitudinal” is used by some to describe the perspective of the anterior‐to‐posterior dimension of the submandibular gland despite imaging with transverse probe placement. Our recommendations are to employ the terms “length” and “depth” when relating the perspective of the gland determined by the transverse placement of the probe.

We recommend reserving the term “longitudinal” to describe probe positioning along the long axis of the patient which is perpendicular to the body of the mandible as is used to assess the “height” of the gland.^38^ A consensus statement from the orthopedic literature identified the correlate of the ultrasound term “longitudinal” to be equivalent to CT/MRI terminology of “coronal” or “sagittal”.^39^ These investigators acknowledged difficulty in finding a consensus in the terminology used to discuss the axes of an isolated structure described out of the context of its relationship to the patient.^39^

**FIGURE 9:**
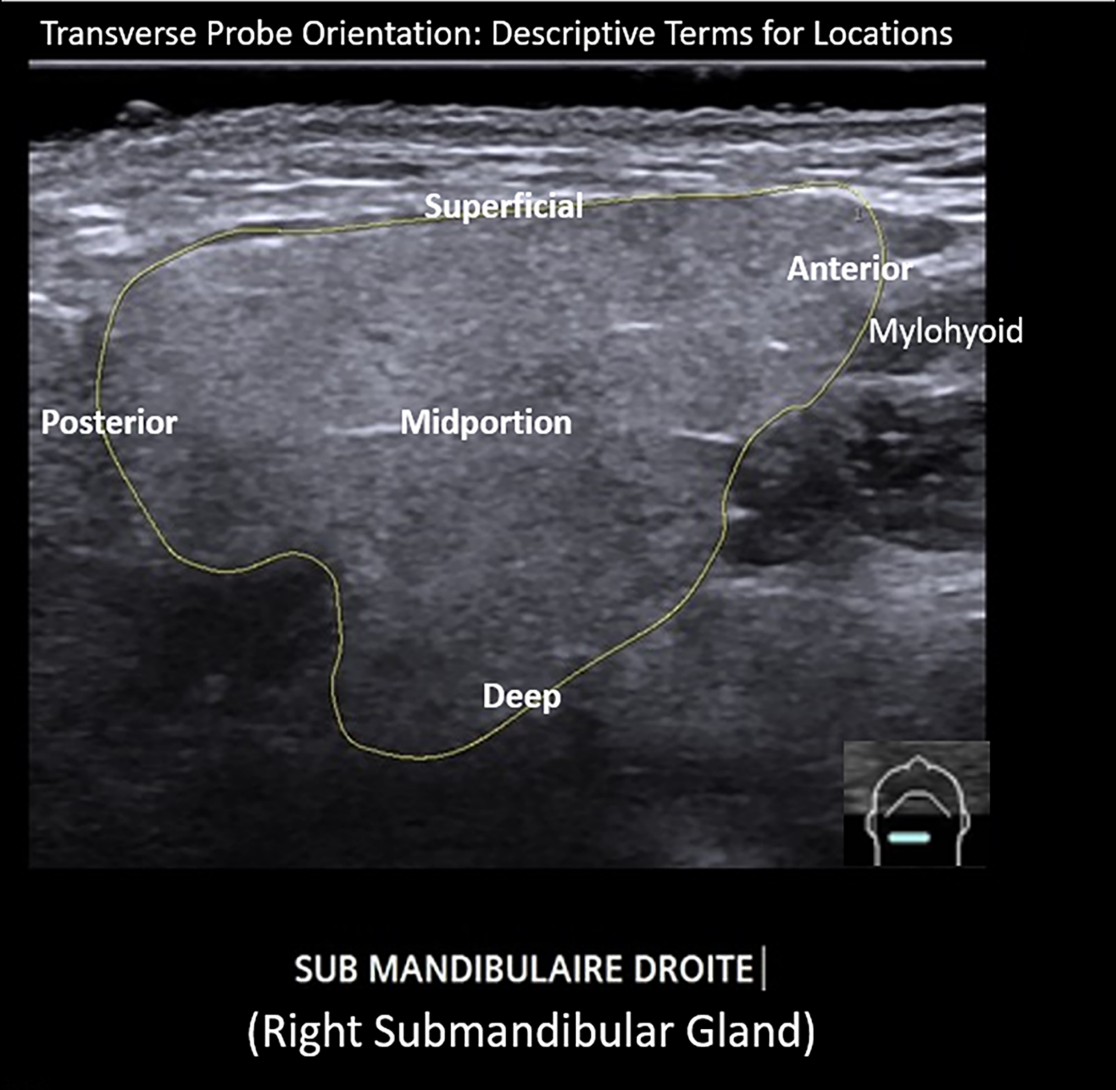
Ultrasound image employing transverse orientation of the probe identifies regions within the outlined right submandibular gland that are labeled with descriptive terms (image courtesy of Dr. Philippe Katz; relabeled).

**FIGURE 10:**
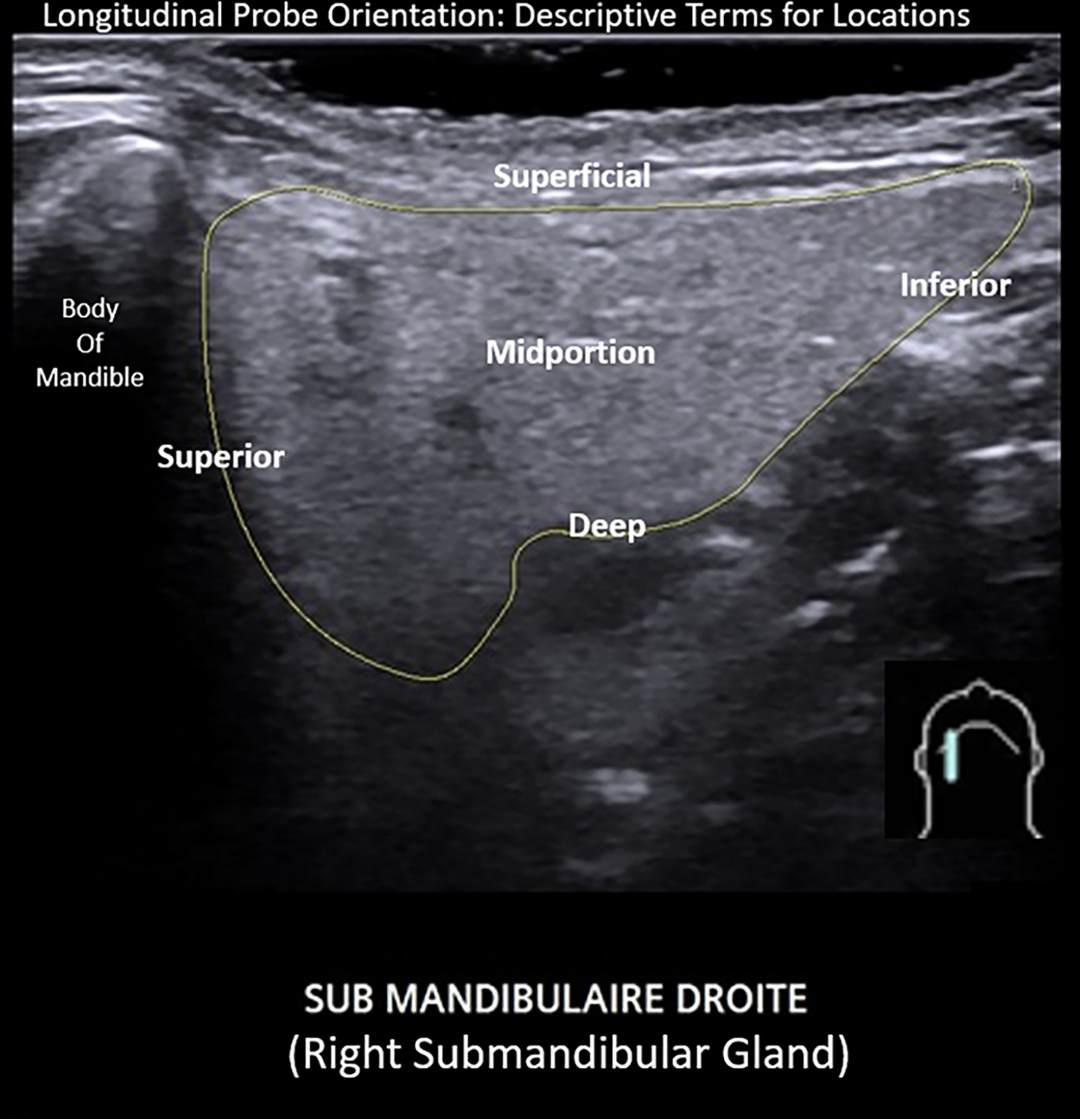
Ultrasound image employing longitudinal orientation of the probe identifies regions within the outlined right submandibular gland that are labeled with descriptive terms (image courtesy of Dr. Philippe Katz; relabeled).

We propose terminology (Table 3) to identify regions within the gland as determined by transverse probe orientation include: “**midportion (or middle)**”, “**anterior**”, “**posterior**”, “**superficial**”, and “**deep**” also depicted in ultrasound examples (Figure 9). Longitudinal orientation of the probe defines “**superior**” and “**inferior**” in addition to “**middle (midportion)**”, “**superficial**” and **“deep”** (Figure 10). As is consistent with measurement of thyroid nodules, we recommend that the dimension of “depth” is reported from measurement employing the transverse but not the longitudinal probe orientation.

**TABLE 3:** Ultrasound Descriptors of Locations within Submandibular Gland.

Ultrasound Probe Orientation	Ultrasound Description of Locations within Submandibular Gland
Transverse	**Anterior**/**Posterior *[Length]***
Longitudinal	**Superior**/**Inferior *[Height]***
Transverse or Longitudinal	**Midportion** (Middle) *Superficial/**Deep** [*Depth] *Reporting of “**Depth”** (the superficial to deep measurement) is limited to transverse probe orientation.

Additional descriptive terminology identifying subsites within the submandibular gland and ductal system have been proposed by Goncalves et al to define stone location along the course of the submandibular duct as determined by the anatomic landmarks of the mylohyoid muscle and sublingual gland (Table 4).^40^ These investigators also proposed a similar system for parotid gland stone location to employ the masseter muscle as the dominant landmark when employing ultrasound to assess the ductal system.

**TABLE 4:** Classification system defining stone location in the gland or ductal system.

Submandibular sonographic terminology and landmarks for sialolithiasis	
Intraparenchymal stone	Proximally located in parenchyma
Proximal/Hilar Stone	1 cm proximal to 1 cm distal to the edge of the mylohyoid muscle
Middle third of duct	1 cm distal to the edge of the mylohyoid muscle to the sublingual gland
Distal ductal system (including papillary region)	Adjacent the sublingual gland extending to the papilla

*Note*: (Adapted with approval from Hoffman HT. Iowa head and neck protocols as further adapted from Goncalves et al 2017 https://medicine.uiowa.edu/iowaprotocols/salivary‐ultrasound. Accessed May 24, 2023).

## 3 CONCLUSION

Details of salivary gland anatomy have been determined through cadaveric dissection, review of static imaging (CT/MRI), and analysis of surgical specimens.^41^, ^42^, ^43^, ^44^, ^45^, ^46^, ^47^ Dynamic ultrasound imaging offers an alternate perspective with advantages and limitations that warrant consistency in assessment and reporting.^48^

The value in assessing salivary gland dimensions to help determine the impact of treatment is emphasized in the EULAR Sjogren's Syndrome Disease Activity Index (ESSDAI) as reported in 2010.^49^ This index provides a score to quantify disease activity and includes salivary gland size as one of several domains evaluated. Ultrasound—in the absence of overlying barriers such as facial hair or soft tissue changes (including obesity)—can image the full extent of the submandibular gland parenchyma to assess size.^27^ Our effort to standardize nomenclature and assessment techniques will hopefully improve upon the vague approaches previously in common use as described by Badarinza wherein the salivary gland size is “*frequently approximated according to intuition and bilateral comparison*”.^50^

Shear wave elastography has become standard in the assessment of liver fibrosis to revolutionize the diagnostic approach to cirrhosis and has markedly diminished the need for liver biopsy.^51^ Similar application of elastography to salivary gland assessment for both neoplastic and non‐neoplastic processes warrants identification of specific sites within the gland in acknowledging that the gland is often affected in a non‐uniform fashion.

Consensus regarding the naming of subsites within the submandibular gland is needed to improve communication about abnormalities detected and to direct the use of more sophisticated assessment schemes such as shear wave analysis targeted to specific regions. Consistency in this terminology should lead to improved reproducibility of findings. Our work to standardize the naming of sites within the submandibular gland is augmented through an ongoing process to address the parotid, sublingual, and minor salivary glands.

The AAO‐HNS Salivary Gland Committee in collaboration with international experts proposes this standardized approach to submandibular gland ultrasound analysis focused on technique and nomenclature.

## CONFLICT OF INTEREST STATEMENT

Hoffman H: Henry T. Hoffman: (a) COOK Medical: Research consultant. (b) UpToDate author. Ryan W: scientific advisory boards for Olympus and Rakuten Medical and consultant for C2DX. Donato F: TriSalus advisory board member. No conflicts of interest related to this publication. Bertelli A: Speaker and travel grants for Merck. Anderson C: former compensated now uncompensated since 2013 consultant for Galera Therapeutics since 2013: research funding from Soligenix; research funding from Galera Therapeutics.

## Supporting information


**Data S1.** Supporting Information.

## References

[1] Moore CL , Copel JA . Point‐of‐care ultrasonography. N Engl J Med. 2011;364(8):749‐757. doi:10.1056/NEJMra0909487 21345104

[2] Liao LJ , Wen MH , Yang TL . Point‐of‐care ultrasound in otolaryngology and head and neck surgery: a prospective survey study. J Formos Med Assoc. 2021;120(8):1547‐1553. doi:10.1016/j.jfma.2021.02.021 33775533

[3] Koch M , Sievert M , Iro H , Mantsopoulos K , Schapher M . Ultrasound in inflammatory and obstructive salivary gland diseases: own experiences and a review of the literature. J Clin Med. 2021;10(16):3547. doi:10.3390/jcm10163547 34441850 PMC8397054

[4] Jousse‐Joulin S , D'Agostino MA , Nicolas C , et al. Video clip assessment of a salivary gland ultrasound scoring system in Sjögren's syndrome using consensual definitions: an OMERACT ultrasound working group reliability exercise. Ann Rheum Dis. 2019;78(7):967‐973. doi:10.1136/annrheumdis-2019-215024 31036626

[5] Cornec D , Jousse‐Joulin S , Pers JO , et al. Contribution of salivary gland ultrasonography to the diagnosis of Sjögren's syndrome: toward new diagnostic criteria? Arthritis Rheum. 2013;65(1):216‐225. doi:10.1002/art.37698 23108632

[6] Hočevar A , Bruyn GA , Terslev L , et al. Development of a new ultrasound scoring system to evaluate glandular inflammation in Sjögren's syndrome: an OMERACT reliability exercise. Rheumatology (Oxford). 2022;61(8):3341‐3350. doi:10.1093/rheumatology/keab876 34849616

[7] Tang G , Luo Y , Mo Y , Yao J , Yang H , Hao S . Diagnostic value of ultrasound evaluation of major salivary glands for Sjögren's syndrome based on the novel OMERACT scoring system. Eur J Radiol. 2023;162:110765. doi:10.1016/j.ejrad.2023.110765 36893528

[8] Jia Y , Yang J , Zhu Y , et al. Ultrasound‐based radiomics: current status, challenges and future opportunities. Med Ultrason. 2022;24(4):451‐460. doi:10.11152/mu-3248 34762720

[9] Salaffi F , Argalia G , Carotti M , Giannini FB , Palombi C . Salivary gland ultrasonography in the evaluation of primary Sjögren's syndrome. Comparison with minor salivary gland biopsy. J Rheumatol. 2000;27(5):1229‐1236. 10813292

[10] Milic VD , Petrovic RR , Boricic IV , et al. Major salivary gland sonography in Sjögren's syndrome: diagnostic value of a novel ultrasonography score (0‐12) for parenchymal inhomogeneity. Scand J Rheumatol. 2010;39(2):160‐166. doi:10.3109/03009740903270623 20059370

[11] Zhang X , Zhang S , He J , et al. Ultrasonographic evaluation of major salivary glands in primary Sjögren's syndrome: comparison of two scoring systems. Rheumatology (Oxford). 2015;54(9):1680‐1687. doi:10.1093/rheumatology/kev103 25936787

[12] Elbeblawy YM , Eshaq Amer Mohamed M . Strain and shear wave ultrasound elastography in evaluation of chronic inflammatory disorders of major salivary glands. Dentomaxillofac Radiol. 2020;49(3):20190225. doi:10.1259/dmfr.20190225 31770001 PMC7068081

[13] Martel A , Coiffier G , Bleuzen A , et al. What is the best salivary gland ultrasonography scoring methods for the diagnosis of primary or secondary Sjögren's syndromes? Joint Bone Spine. 2019;86(2):211‐217. doi:10.1016/j.jbspin.2018.06.014 30053612

[14] AIUM . Standard Presentation and Labeling of Ultrasound Images. 6th ed. American Institute of Ultrasound in Medicine; 2020. Available at: http://aium.s3.amazonaws.com/resourceLibrary/splv6.pdf Accessed May 10, 2023.

[15] Hofauer B , Roth A , Heiser C , et al. Point shear wave elastography in diagnosis and follow‐up of salivary gland affection after head and neck cancer treatment. J Clin Med. 2022;11(21):1‐12. doi:10.3390/jcm11216285 PMC9654639 36362513

[16] Knopf A , Hofauer B , Thürmel K , et al. Diagnostic utility of acoustic radiation force impulse (ARFI) imaging in primary Sjoegren's syndrome. Eur Radiol. 2015;25(10):3027‐3034. doi:10.1007/s00330-015-3705-4 25861884

[17] Bamber J , Cosgrove D , Dietrich CF , et al. EFSUMB guidelines and recommendations on the clinical use of ultrasound elastography. Part 1: basic principles and technology. Ultraschall Med. 2013;34(2):169‐184. doi:10.1055/s-0033-1335205 23558397

[18] Shiina T , Nightingale KR , Palmeri ML , et al. WFUMB guidelines and recommendations for clinical use of ultrasound elastography: part 1: basic principles and terminology. Ultrasound Med Biol. 2015;41(5):1126‐1147. doi:10.1016/j.ultrasmedbio.2015.03.009 25805059

[19] Hetzel G , Lang W , Strobel D , Iro H , Bozzato A , Zenk J , eds. Atlas of Head and Neck Ultrasound. Georg Thieme Verlag KG; 2013. doi:10.1055/b-002-91659

[20] Ugalde P , Camargo Jde J , Deslauriers J . Lobes, fissures, and bronchopulmonary segments. Thorac Surg Clin. 2007;17(4):587‐599. doi:10.1016/j.thorsurg.2006.12.008 18271171

[21] Bismuth H . Revisiting liver anatomy and terminology of hepatectomies. Ann Surg. 2013;257(3):383‐386. doi:10.1097/SLA.0b013e31827f171f 23386236

[22] Juza RM , Pauli EM . Clinical and surgical anatomy of the liver: a review for clinicians. Clin Anat. 2014;27(5):764‐769. doi:10.1002/ca.22350 24453062

[23] Garcia‐Serrano G , Moñux A , Maranillo E , et al. Vascular clinical anatomy of the submandibular gland. J Craniomaxillofac Surg. 2020;48(6):582‐589. doi:10.1016/j.jcms.2020.04.004 32389551

[24] Yazbeck A , Iwanaga J , Walocha JA , Olewnik Ł , Tubbs RS . The clinical anatomy of the accessory submandibular gland: a comprehensive review. Anat Cell Biol. 2023;56(1):9‐15. doi:10.5115/acb.22.118 36384887 PMC9989781

[25] O'Daniel TG . Understanding deep neck anatomy and its clinical relevance. Clin Plast Surg. 2018;45(4):447‐454. doi:10.1016/j.cps.2018.06.011 30268237

[26] Hocevar A , Ambrozic A , Rozman B , Kveder T , Tomsic M . Ultrasonographic changes of major salivary glands in primary Sjogren's syndrome. Diagnostic value of a novel scoring system. Rheumatology (Oxford). 2005;44(6):768‐772. doi:10.1093/rheumatology/keh588 15741192

[27] Badarinza M , Serban O , Maghear L , et al. Multimodal ultrasound investigation (grey scale, doppler and 2D‐SWE) of salivary and lacrimal glands in healthy people and patients with diabetes mellitus and/or obesity, with or without sialosis. Med Ultrason. 2019;21(3):257‐264. doi:10.11152/mu-2164 31476205

[28] Leppi TJ . Gross anatomical relationships between primate submandibular and sublingual salivary glands. J Dent Res. 1967;46(2):359‐365. doi:10.1177/00220345670460020801 4960427

[29] Katz P , Hartl DM , Guerre A . Clinical ultrasound of the salivary glands. Otolaryngol Clin N Am. 2009;42(6):973‐1000, Table of Contents. doi:10.1016/j.otc.2009.08.009 19962004

[30] Li L , Gao XL , Song YZ , et al. Anatomy of arteries and veins of submandibular glands. Chin Med J. 2007;120(13):1179‐1182. 17637249

[31] AIUM‐ACR‐SPR‐SRU Practice Parameter for the Performance and Interpretation of a Diagnostic Ultrasound Examination of the Extracranial Head and Neck. J Ultrasound Med. 2018;37(11):E6‐e12. doi:10.1002/jum.14830 30308087

[32] Patel NJ , Hashemi S , Joshi AS . Sonopalpation: a novel application of ultrasound for detection of submandibular calculi. Otolaryngol Head Neck Surg. 2014;151(5):770‐775. doi:10.1177/0194599814545736 25091191

[33] Bahner DP , Blickendorf JM , Bockbrader M , et al. Language of transducer manipulation: codifying terms for effective teaching. J Ultrasound Med. 2016;35(1):183‐188. doi:10.7863/ultra.15.02036 26679204

[34] Stevenson JH . Tissue scanning. In: Daniels JM , Dexter WW , eds. Basics of Musculoskeletal Ultrasound. Springer; 2013:15‐27.

[35] Blessing M . Ultrasound Probe Selection, Knobology and Optimization of Image Quality. In: Li J , Ming‐Der Chow R , Vadivelu N , Kaye AD , eds. Ultrasound Fundamentals: An Evidence‐Based Guide for Medical Practitioners. Springer International Publishing; 2021:17‐24.

[36] Situ‐LaCasse E , Acuña J . Principles of ultrasound guidance. In: Adhikari S , Blaivas M , eds. The Ultimate Guide to Point‐of‐Care Ultrasound‐Guided Procedures. Springer International Publishing; 2020:5‐27.

[37] Etter LE . Glossary of Words and Phrases Used in Radiology, Nuclear Medicine, and Ultrasound: Prepared from Various Sources for Medical Secretaries, X‐Ray Technicians, Medical Students, and Residents in Radiology. C.C. Thomas; 1970.

[38] Hoffman HT , Pagedar NA . Ultrasound‐guided salivary gland techniques and interpretations. Atlas Oral Maxillofac Surg Clin North Am. 2018;26(2):119‐132. doi:10.1016/j.cxom.2018.04.001 30077320

[39] Hall MM , Allen GM , Allison S , et al. Recommended musculoskeletal and sports ultrasound terminology: a Delphi‐based consensus statement. J Ultrasound Med. 2022;41(10):2395‐2412. doi:10.1002/jum.15947 35103998

[40] Goncalves M , Schapher M , Iro H , Wuest W , Mantsopoulos K , Koch M . Value of sonography in the diagnosis of sialolithiasis: comparison with the reference standard of direct stone identification. J Ultrasound Med. 2017;36(11):2227‐2235. doi:10.1002/jum.14255 28556090

[41] Davis RA , Anson BJ , Budinger JM , Kurth LR . Surgical anatomy of the facial nerve and parotid gland based upon a study of 350 cervicofacial halves. Surg Gynecol Obstet. 1956;102(4):385‐412. 13311719

[42] Brouwer CL , Steenbakkers RJ , Bourhis J , et al. CT‐based delineation of organs at risk in the head and neck region: DAHANCA, EORTC, GORTEC, HKNPCSG, NCIC CTG, NCRI, NRG Oncology and TROG consensus guidelines. Radiother Oncol. 2015;117(1):83‐90. doi:10.1016/j.radonc.2015.07.041 26277855

[43] Paczona VR , Capala ME , Deák‐Karancsi B , et al. Magnetic resonance imaging‐based delineation of organs at risk in the head and neck region. Adv Radiat Oncol. 2023;8(2):101042. doi:10.1016/j.adro.2022.101042 36636382 PMC9830100

[44] Afzelius P , Nielsen MY , Ewertsen C , Bloch KP . Imaging of the major salivary glands. Clin Physiol Funct Imaging. 2016;36(1):1‐10. doi:10.1111/cpf.12199 25319072

[45] Quer M , Guntinas‐Lichius O , Marchal F , et al. Classification of parotidectomies: a proposal of the European salivary gland society. Eur Arch Otorhinolaryngol. 2016;273(10):3307‐3312. doi:10.1007/s00405-016-3916-6 26861548

[46] Pujol‐Olmo A , Mirapeix RM , Sañudo‐Tejero JR , Quer‐Agustí M . Description and relationships of the parotid gland levels proposed by the European salivary gland society staging system: an anatomical study. Surg Radiol Anat. 2020;42(9):1101‐1107. doi:10.1007/s00276-020-02483-x 32372113

[47] Abdalla‐Aslan R , Keshet N , Zadik Y , Aframian DJ , Nadler C . Standardization of terminology, imaging features, and interpretation of CBCT sialography of major salivary glands: a clinical review. Quintessence Int. 2021;52(8):728‐740. doi:10.3290/j.qi.b1492217 34076380

[48] Lorenzon M , Spina E , Tulipano Di Franco F , Giovannini I , De Vita S , Zabotti A . Salivary gland ultrasound in primary Sjögren's syndrome: current and future perspectives. Open Access Rheumatol. 2022;14:147‐160. doi:10.2147/oarrr.S284763 36072437 PMC9444027

[49] Seror R , Ravaud P , Bowman SJ , et al. EULAR Sjogren's syndrome disease activity index: development of a consensus systemic disease activity index for primary Sjogren's syndrome. Ann Rheum Dis. 2010;69(6):1103‐1109. doi:10.1136/ard.2009.110619 19561361 PMC2937022

[50] Bozzato A , Burger P , Zenk J , Uter W , Iro H . Salivary gland biometry in female patients with eating disorders. Eur Arch Otorhinolaryngol. 2008;265(9):1095‐1102. doi:10.1007/s00405-008-0598-8 18253742

[51] Tamaki N , Kurosaki M , Huang DQ , Loomba R . Noninvasive assessment of liver fibrosis and its clinical significance in nonalcoholic fatty liver disease. Hepatol Res. 2022;52(6):497‐507. doi:10.1111/hepr.13764 35352460 PMC9718363

