# Supplementary material for: Proposal for standardized ultrasound analysis of the salivary glands: Part 1 submandibular gland
Source: Laryngoscope Investig Otolaryngol. 2024 Feb 14;9(1):e1224. doi: 10.1002/lio2.1224 (PMC10866606; doi:10.1002/lio2.1224)
Supplement: Supplementary file 1 — Data S1. Supporting Information. [file LIO2-9-e1224-s001.docx]

# Supplement to Proposal for Standardized Ultrasound Analysis of the Salivary Glands: Part 1 Submandibular Gland (supplement)

Henry T Hoffman MD University of Iowa *

Michael Koch MD University of Erlangen-Nuremberg, Germany

Robert Lee Witt MD Christiana Care/Thomas Jefferson University

William R. Ryan MD University of California San Francisco *

Johannes Zenk MD University Hospital Augsburg, Germany

Philippe Katz MD Institut d'Explorations Fonctionnelles des Glandes Salivaires, Paris, France

Rahmatullah Rahmati MD Harvard Medical School

Christopher Rassekh MD University of Pennsylvania Perelman School of Medicine

Francisco Donato MD University of Iowa Hospitals and Clinics *

Timothy M McCulloch MD University of Wisconsin School of Medicine and Public Health

Arjun S Joshi MD The George Washington University

Jolie Lien Chang MD University of California, San Francisco

M. Boyd Gillespie MD University of Tennessee Health Science Center College of Medicine

Priscilla F. A. Pichardo DO Geisinger Health System

Lisa Ann Orloff MD Stanford University

Antoino Marcelino MD University of Iowa Hospitals and Clinics

Piper Wenzel BS University of Iowa Hospitals and Clinics

David Cohen MD Kaiser Permanente Los Angeles Medical Center

Christopher E Fundakowski MD Thomas Jefferson University Hospital

David M Cognetti MD Thomas Jefferson University Hospital,

Rohan R Walvekar MD Louisiana State University HSC

Antonio Bertelli Faculdade de Ciencias Medicas da Santa Casa de Sao Paulo Brazil *

Harry Quon MD Johns Hopkins Medical Institutions Campus

Carryn Anderson MD University of Iowa Hospitals and Clinics *

Bruno Policeni MD University of Iowa Hospitals and Clinics

Gordy Siegel MD Northwestern University Feinberg School of Medicine

**Introduction**

This supplement to the more concise proposal to standardize ultrasound assessment provides a detailed literature review along with illustrative case examples.

Salivary gland anatomy have been determined through cadaveric dissection, review of static imaging (CT/MRI), and analysis of surgical specimens.^1-7^ Dynamic ultrasound imaging offers an alternate perspective with advantages and limitations that warrant guidelines to provide consistency in assessment and reporting.^8^

**Biometry** describes the “*measurement … of living tissue or bodily structures*”.^9^ Assessment of the size of an organ through three-dimensional reconstruction may be accomplished through CT and MR imaging. Clinical application of this technique is applied by radiation oncologists through the radiographic ‘*contouring*’ of organs in designing treatment plans.^10,11^

Ultrasound assessment of submandibular size usually does not include consideration of the anterior extension of Wharton’s duct with surrounding tissue which, despite use of sonopalpation, can be difficult to image due to shielding from bone of the mandible.^12^ Ultrasound - in the absence of overlying barriers such as facial hair or soft tissue changes (including obesity) - can usually image the parenchyma of the submandibular gland to assess size.^13^ However, 2D ultrasound, available in most diagnostic practices, is less capable of quantifying the volume of the irregularly shaped salivary glands due to the inability to analyze the aggregate dimensions of multiple thin slices as is performed with computed tomography.^14^ As a result, salivary gland size is “*frequently approximated according to intuition and bilateral comparison*”.^15^ Efforts to quantify salivary gland size by ultrasound have addressed these shortcomings through measurements described with inconsistent use of terminology including ’*length*’, ‘*depth*’, ‘*thickness*’, ‘*width*’ and ‘*height*’.^16-20^

**Radiomics** is broadly defined as a method to extract information from medical images and has received intense scrutiny with CT and MRI. It is reported that the analysis of ‘*radiomic features”* has the *‘potential to uncover patterns and characteristics that fail to be appreciated by the naked eye”* which are *“useful to predicting prognosis and therapeutic response”*.^21^ Advances in image processing, informatics, and machine learning have led to the concept of a ‘*radiomic signature*’ to characterize anatomic structures and tumors.^10^ This signature provides an image guided ‘*textural analysis*’ employing ‘*image biomarkers*.’^22,23^ One focus of this type of work has been to address changes to the radiomic signature of salivary glands occurring during radiotherapy to correlate with the development of xerostomia.^10^

Ultrasound radiomics is a developing field with acknowledged limitations due to inconsistency in image acquisition and difficulties in calibrating quantitative methods.^24^ Subjective grading of salivary ultrasound images through semi-quantitative classification schemes has been supplemented by quantitative assessment employing shear wave elastography to further refine analysis.^25-29^ Assessments by both approaches generally lack consistency in identifying specific anatomic regions (subsites) within the salivary glands and rarely report differences between regions in the same gland.^30,31^

Shear wave elastography is a quantitative ultrasound method to determine the velocity of tissue displacement resulting from a secondary ‘push pulse’ produced by the ultrasound probe. The speed of tissue displacement (shear wave) correlates with the tissue stiffness or fibrosis from the selected “regions of interest” evaluated.^32^ Identifying the specific “regions of interest” (subsites) assessed within the salivary glands is needed to provide consistency in these shear wave measurements.^33-36^

This report addresses the standardization of salivary gland ultrasound nomenclature and measurement technique with additional focus on the reporting to include specific subsites within the gland. Recommendations are designed to provide consistency in salivary gland imaging intended for clinical and research applications within the evolving field of ultrasound radiomics.^37^

This detailed supplement provides additional insight addressing recommended terminology and technique. This work derives from the Salivary Gland Committee of the AAO-HNS supplemented by additional expertise to provide the first in a series of proposals for to standardize salivary gland assessment and reporting, with this initial report addressing the submandibular gland.

# Background

Analysis of outcomes from salivary gland treatment employing ultrasound requires the use of reproducible imaging techniques.^38^ Treatments currently under study range from botulinum toxin injection to medical therapy for autoimmune sialadenitis to surgical treatment to relieve duct obstruction and include investigative work such as salivary duct infusion (drug / gene therapy) as well as parenchymal injection (stem cell injection) to address xerostomia.^39-46^

The value in assessing salivary gland dimensions to help determine the impact of treatment is emphasized in the EULAR (European League Against Rheumatism) Sjogren’s Syndrome Disease Activity Index (ESSDAI) as reported in 2010.^47^ This index provides a score to quantify disease activity and includes salivary gland size as one of several domains evaluated. These investigators define parotid gland enlargement as greater than 3 cm without specifying the technique used to determine gland size. Submandibular gland assessment is even less well characterized. These investigators used the label ‘*important submandibular enlargement*’ to define size abnormality without addressing how the ‘*enlargement*’ is determined.

An updated 2017 publication from EULAR presented a consensus addressing ultrasound evaluation for 9 separate anatomic sites, including the salivary glands.^48^ This consensus statement related that experience in the *'evaluation of salivary glands was limited*’ and ‘*standardization of the scanning procedures for these structures would further facilitate their clinical application and encourage further research.*’

Consensus regarding the naming of subsites within the submandibular gland is needed to improve communication about abnormalities detected and to direct the use of more sophisticated assessment schemes such as shear wave analysis targeted to specific regions. Consistency in the nomenclature used to identify locations within these subsites should lead to improved reproducibility of findings.

# Submandibular Gland Anatomy and Subsites

The submandibular gland shape may be modified by tumor, ductal obstruction, atrophy, inflammation, trauma, response to medical therapy and irradiation, or a combination of all these factors. Additionally, in the normal state, the salivary glands are dynamic structures whose blood flow, secretory status, size, and location have been reported to change according to circadian rhythms, degree of hydration, gustatory stimulation, and mechanical movement associated with adjacent muscular forces.^49,50^ Gland size has also been reported to vary according to the degree of fatty infiltration as influenced by factors such as age, diabetes, and other systemic metabolic disorders.^20,51,52^ Ambient temperature and psychological stress are other factors reported to influence salivary gland characteristics.^25^

The normal shape of the submandibular gland is broadly considered ellipsoid despite the description in the 1985 version of Gray’s Anatomy as ‘*round and about the size of a walnut*’.^53^ Gray’s text describes the gland as having a superficial and a deep surface but isolates only a single subunit termed a ‘deep process’ of the gland extending anteriorly between the mylohyoid muscle laterally and the hyoglossus and styloglossus muscles medially. Other anatomists identify this extension as the ‘*deep portion of the gland typically folded around the posterior edge of the [mylohyoid] muscle*.’^54^ This deep portion where the gland ‘*wraps around the posterior edge of the mylohyoid muscle*’ has also been termed ‘*the uncinate process’* or ‘*the deep arm*’ of the submandibular gland.^55,56^

Saban et al divided the gland into ‘*two sets of lobes: superficial and deep*”.^57^ These investigators identified the paired submandibular glands as being like “*elongated, flattened hooks”* with the deep lobe hooking around the posterior margin of the mylohyoid muscle. They compared the submandibular gland to a “*triangular almond*” whose anterior border is divided by the mylohyoid muscle to define the two lobes – superficial and deep. Although multiple investigators use the mylohyoid to distinguish the superficial lobe (superficial - under the skin) from the deep lobe (above the mylohyoid), these assessments fail to define the extent of separation into lobes posterior to the free edge of the mylohyoid.^58,59^ Additionally, the terminology is not standardized to describe subsites within the lobes of the gland.

We propose that the submandibular gland is comprised of a superficial lobe and a deep lobe. The deep lobe also includes an anterior projection termed the uncinate process partially surrounding Wharton’s duct. The traditional use of the term ‘lobe’ is clearly defined when applied to the lung to define aggregates of bronchopulmonary segments determined by bronchial branching.^60^ The pulmonary lobes are also identifiable through the classic definition that “a lobe is a part of an organ defined by a fissure seen at the surface of the organ”.^61^

The superficial ‘lobe’ of the submandibular gland is more accurately termed the superficial ‘aspect’ or ‘portion’ in that discrimination from the deep ‘aspect’ of the gland is determined by relationship to an external structure (the mylohyoid muscle) and not internal organization or an identifiable fissure. In a similar manner, the liver has been broadly classified as having right and left lobes that do not correspond to the more critical subdivision into multiple sectors or segments discriminated by blood supply and biliary flow.^62^ We acknowledge the more accurate division of the submandibular gland into two “aspects” – the superficial and deep portions. However, due to established convention, the more widely used terminology employing the terms ‘lobes’ is also acceptable.

Also considered within the anatomic unit of the submandibular gland are both the surrounding capsule as well as vascular structures where these vessels are surrounded by gland parenchyma (**Table 1**).^63^ The capsule of the submandibular gland has been identified as a continuation of the investing layer of the deep cervical fascia.^64^ As per O’Daniel, this fascia is thicker anteriorly (superficial aspect) and thinner posteriorly (deep aspect).^65^ A normal capsule to the submandibular gland will be identified with a hyperechoic appearance – with loss of definition of the gland border considered a sufficiently abnormal finding that it has been identified in ultrasound grading scales to support the diagnosis of Sjogren’s syndrome.^28,31,66^ Obesity, diabetes and sialosis have also been identified to be associated with an invisible deep (posterior) border.^13,67^

The superficial lobe is distinguished from the deep lobe by a line running parallel to the transverse axis of the gland as determined by the anterior margin of the mylohyoid muscle (**Figure 1**).

The deep lobe includes the uncinate process, which is defined as the anterior extension of the submandibular gland between the mylohyoid and hyoglossus muscles (**Figure 2**).

This uncinate process of the submandibular gland includes parenchymal tissue surrounding Wharton’s duct and may be difficult to discriminate on ultrasound examination from sublingual gland tissue. Leppi performed elegant cadaveric dissections to discriminate the uncinate process from the sublingual gland and identified ‘variable groupings’ of submandibular tissue above the mylohyoid muscle **(Figure 3)**.^68^ Although he termed these variable anterior extensions of the submandibular gland as ‘accessory glands’ and ‘secondary glands’, we feel the term ‘uncinate process’ of the submandibular gland most effectively describes this anatomic extension.

The anatomy of the floor of mouth is complicated by the variable relationships not only between the sublingual gland and Whartons duct but also by variety in the extent of the uncinate process (**Figure 4**).

The mylohyoid muscle serves as a reference in assessing the relationship between the uncinate process and the sublingual gland as highlighted in an example showing a plunging ranula protruding through a dehiscence in the mylohyoid muscle (**Figure S1**)

**Figure S1** Ultrasound (14-5 MHz linear probe transverse) identifying the uncinate process abutting the sublingual gland further defined by a dehiscence in the mylohyoid muscle with protrusion of the sublingual gland communicating with a plunging ranula. (with approval from Hoffman HT (ed) Iowa Head and Neck Protocols <[Submandibular Gland Anatomy: The Uncinate Process of the Deep Lobe | Iowa Head and Neck Protocols (uiowa.edu)](https://medicine.uiowa.edu/iowaprotocols/submandibular-gland-anatomy-uncinate-process-deep-lobe) > accessed April 2, 2023


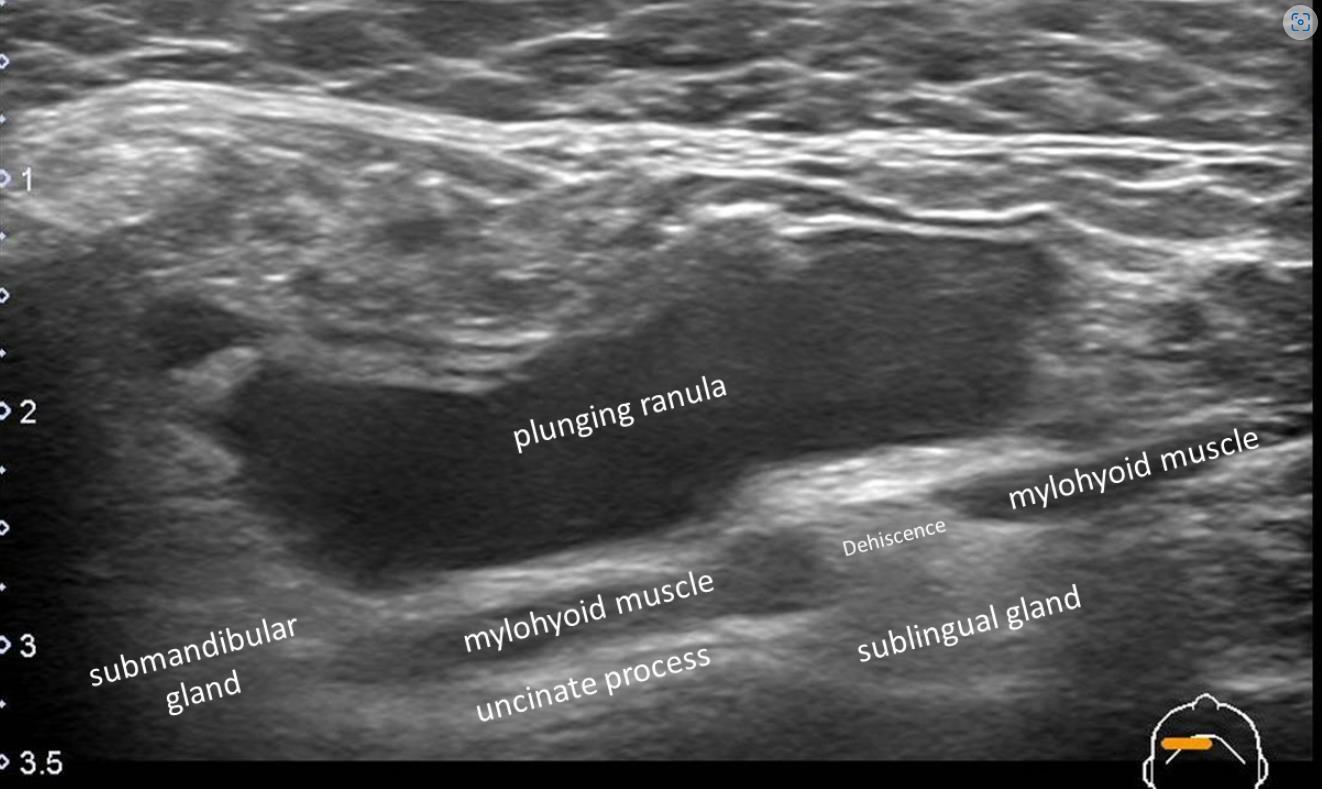


Anatomic variation to the posterior and superior aspects of the submandibular gland may create difficulty in measuring the extent of parenchyma due to variability in the vascular structures in this region. Ultrasound with color doppler is useful not only in discriminating between blood vessels and non-vascular ductal elements but may direct more accurate size measurement of the submandibular gland by identifying where blood vessels are surrounded by gland parenchyma **(Figure 5 and S2).**^67,69^

Although the arterial blood supply to the submandibular gland may also arise from the lingual, deep lingual, and external carotid arteries, the dominant blood supply is from the facial artery (including the submental branch of the facial artery). Li et al identified that the facial artery runs along a ‘*groove*’ within the submandibular gland and can be surrounded by the cortex of the gland.^69,70^

Venous drainage from the submandibular gland has been termed ‘*complicated and variable*’ but is primarily ascribed to the “*anterior facial vein, hilum vein of the gland, and the venae commitantes of the facial artery*”.^70^ Other vascular drainage includes the mental vein, deep lingual vein, external jugular vein and anterior jugular vein. An understanding of this variable venous drainage of the submandibular gland gained increased attention coordinate with the expanded use of transplanted vascularized autologous submandibular glands in the management of keratoconjunctivitis sicca.^71,72^

Ultrasound with color Doppler is capable of dynamically identifying the blood vessels that are included within and may permit more accurate gland size measurement.

**Figure S2** Left submandibular gland resection identifying the facial blood vessels in a deep groove in the posterior gland warranting removal of the gland in two pieces in order to preserve the facial artery.

(with approval from Hoffman HT (ed) Iowa Head and Neck Protocols <Submandibular Gland Anatomy:

Vascular Supply - Ultrasound Imaging with Color Doppler

https://medicine.uiowa.edu/iowaprotocols/submandibular-gland-anatomy-vascular-supply-ultrasoundimaging-color-doppler> accessed June 18, 2023)


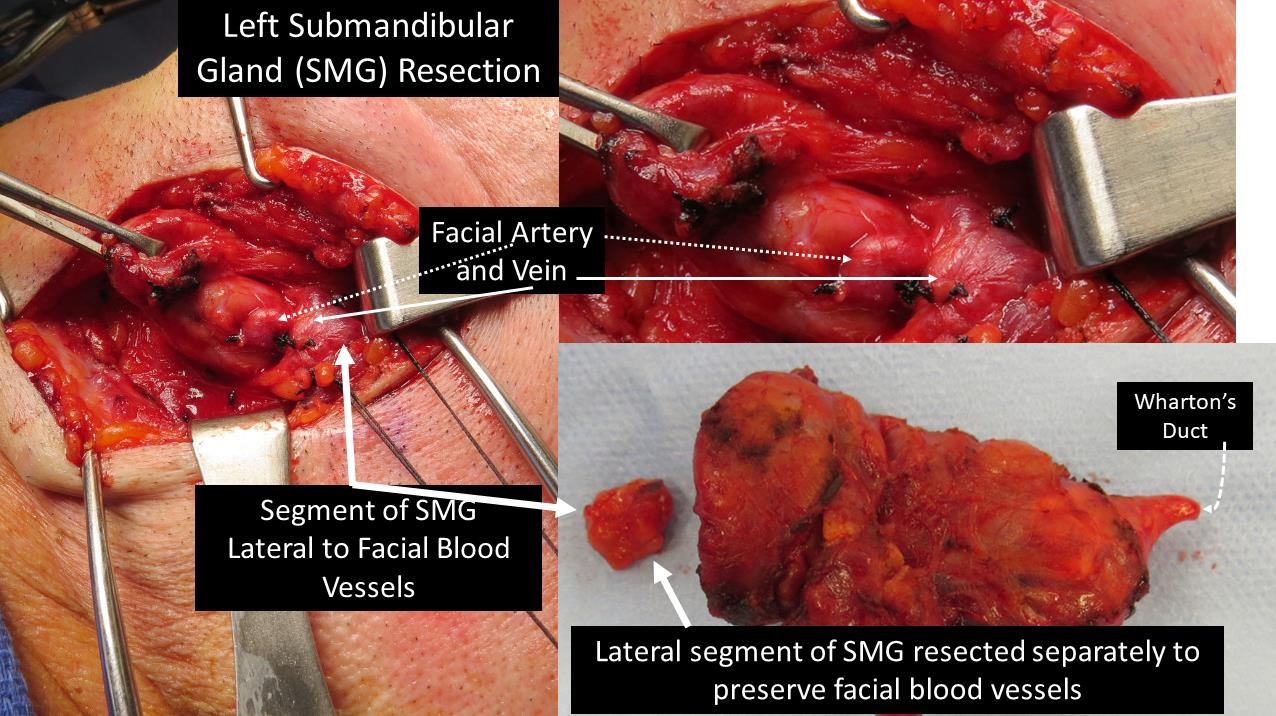


# Salivary Ultrasound Assessment Techniques

## Equipment

As is recommended by the American Institute of Ultrasound Medicine (AIUM) for “extracranial head and neck ultrasound evaluations”, we support use of a linear transducer for salivary gland evaluation.^73^ The AIUM recommends a mean frequency of 10 to 14 MHz probe and notes that a greater depth of penetration may warrant use of lower frequencies.

## Technique

Submandibular gland size determined by simple palpation was found to be inadequate when compared to that provided by ultrasound analysis as reported by Marteau et al.^74^ These investigators suggested the submandibular gland length to be assessed by ultrasound in the transverse axis below the inferior border of the mandible. Ultrasound determination of the width of the gland was done by ‘*begirdin*g’ the gland to provide an estimate of the surface area calculated by the formula: (length x width)/2. The classification of ‘*ultrasound hypertrophy*’ assigned to submandibular glands was given if greater than 3 cm^2^.

Dost and Kaiser in 1997 reported comparisons of submandibular gland volumes determined by ultrasound sound analysis in 50 normal subjects stratified by age and gender.^18^ The submandibular glands were scanned at an angle ‘*to visualize as much of the gland as possible*’ to include 3 dimensions: anterior-posterior, lateral-medial, and depth. The orientation of the probes was described as placed in the **‘*paramandibular plane*’** and ‘***frontal plane***’ (**Figure S3**). These investigators reported a poor correlation when comparing ultrasound volume measurement (using Simpson’s formula) of cadaveric submandibular glands (fixed with formalin) with additional measurement by water displacement.

**Figure S3** Ultrasonographic scanning planes for submandibular gland exam. P = paramandibular plane, f = frontal plane "Reprinted from Ultrasound in Medicine & Biology, Vol 23, No.9 Dost P and Kaiser S: Ultrasonographic Biometry in Salivary Glands Pages pp 1299-1303 Copyright (1997), with permission from Elsevier. (License number 5473311090067)"


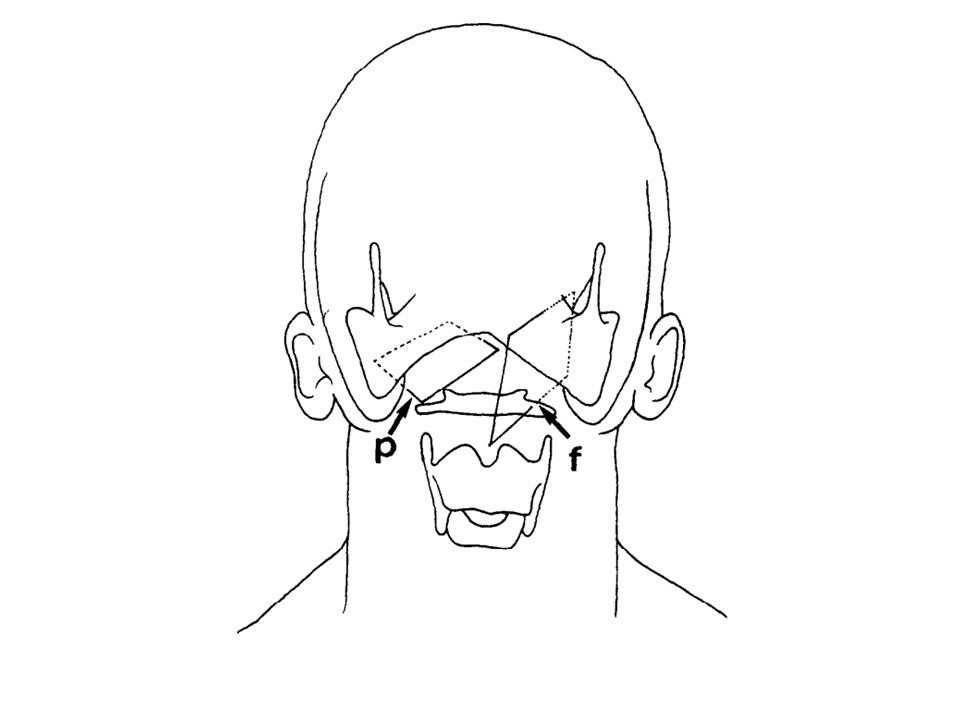


A recent study by Açıkgöz et al employed a linear 12-MHz transducer probe to examine the submandibular glands in 2 perpendicular planes that approximated the transverse and longitudinal orientation.^19^ Similar to Dost and Kaiser, these investigators described probe orientation as ‘parallel to the lower border of the mandible’ (approximating transverse orientation) and ‘vertical to the mandible body” (longitudinal) as a technique also supported by others.^75^

Açıkgöz et al measured 3 dimensions from these evaluations and selected the greatest size labelled as:^19^

1. “*Length*” = “Anteroposterior length” (transverse orientation)
2. “*Height*” = “Superoinferior length” (transverse orientation)
3. “*Width or thickness*” = “Mediolateral length” (vertical orientation)

These three dimensions were then used to calculate volume by formula assuming ellipsoid three-dimensional shape to submandibular gland.

Although the terms ‘transverse’ and ‘longitudinal’ may be used without modifiers, some degree of obliquity relative to the central axis is usually introduced to permit examination with the probe parallel (transverse) and perpendicular (longitudinal) to the body of the mandible usually initiated perpendicular to the skin surface (**Figure 8**)

Additional imaging with the probe position altered from this initial orientation is generally required to identify ductal and hilar stones.^76^ Angling the transversely positioned probe in a rostral direction (under the mandible) is often needed and may be supplemented by intraoral digital depression of the floor of the mouth to deliver structures into the field of view by the process termed ‘sono-palpation’.

The American Institute of Ultrasound in Medicine (AIUM) has published practice parameters for the documentation of an ultrasound examination.^73^ These generalized guidelines emphasize that ‘*accurate and complete documentation and communication are essential for high-quality patient care*”. Recording of anatomic measurements is recommended when appropriate. A separate publication from the AIUM addressing head and neck ultrasound offers more specific recommendations to identify that reporting of ‘*focal abnormalities within the salivary glands should include the size in 3 dimensions*”.^32^

We propose terminology to standardize assessment and reporting of ultrasound probe positioning and assessment (**Table 2**) highlighted by representative ultrasound images (**Figures 6 and 7**). Ultrasound assessment of submandibular size usually does not include consideration of the anterior extension of Wharton’s duct with surrounding tissue. The anterior extension of Wharton’s duct can occasionally be difficult to image due to shielding from bone of the mandible despite use of sonopalpation.^12^

An ellipsoid model to calculate the ultrasound-determined volume of the thyroid gland (width x length x thickness x 0.52) was proposed by Rago et al and further modified to analyze submandibular gland volume by Manetti et al.^17,77^ Through this approach, Manetti et al reported submandibular gland volumes to range from 4.6 to 12.9 ml in 200 normal subjects with no differences identified for age, gender or BMI. A volume of 13 ml was considered the upper normal limit. They affirmed that ‘*ultrasound allows an estimate of submandibular salivary gland biometry and calculation of the exact gland volume by the ellipsoid formula*’. It is noteworthy that these calculations must be considered an approximation due to deviations of most submandibular glands from the presumed ellipsoid shape - including the presence of an uncinate process.

The terminology addressing probe orientation is confusing and differs based on whether the long axis of the body or the long axis of the structure studied is emphasized.^78^ The term ‘transverse’ is recommended to describe imaging with the probe positioned parallel to the body of the mandible – similar to ‘axial’ or ‘cross-sectional’ - in a plane that is perpendicular to the long axis of the body.^79-81^ Differences persist in the literature regarding terminology to description the image of the gland as identified by this transverse probe placement. The word ‘longitudinal’ is used by some to describe the perspective of the anterior-to-posterior dimension of the submandibular gland despite imaging with transverse probe placement. Our recommendations are to employ the terms 'length’ and “depth” when relating the perspective of the gland determined by the transverse placement of the probe.

We recommend reserving the term ‘longitudinal’ to describe probe positioning along the long axis of the body which is perpendicular to the body of the mandible as is used to assess the ‘height’ of the gland.^82^ A consensus statement in the orthopedic literature identified the correlate of the ultrasound term ‘longitudinal’ to be equivalent to CT/MRI terminology of ‘coronal’ or ‘sagittal’.^83^ These investigators acknowledged difficulty in finding a consensus in the terminology used to discuss the axes of an isolated structure described out of the context of its relationship to the body.^83^

We propose terminology **(Table 3)** to identify regions within the gland as determined by transverse probe orientation include: “**midportion (or middle)**”, “**anterior**”, “**posterior**”, “**superficial**”, and “**deep**” also depicted in ultrasound examples (**Figure 9**). Longitudinal orientation of the probe defines “**superior**” and “**inferior**” in addition to “**middle (midportion)**”, ‘**superficial**’ and **‘deep’** (**Figure 10**). As is consistent with measurement of thyroid nodules, we recommend that the dimension of ‘depth’ is reported from measurement employing the transverse but not the longitudinal probe orientation.

Additional descriptive terminology identifying subsites within the submandibular gland and ductal system have been proposed by Goncalves et al to define stone location along the course of the submandibular duct as determined by the anatomic landmarks of the mylohyoid muscle and sublingual gland (**Table 4**).^84^ These investigators also proposed a similar system for parotid gland stone location to employ the masseter muscle as the dominant landmark when employing ultrasound to assess the ductal system.

# Case Examples

## Case #1 Consistency in size assessment – pressure of ultrasound probe placement

The manner by which the ultrasound probe is placed on the skin overlying the region to study influences the characteristics of the tissue evaluated. Assessment should be done with minimal to no pressure by the probe when placed over the region evaluated.

The **depth** of the normal left submandibular gland was measured as 12 mm with light application (no pressure) of the linear 14-5 MHz probe positioned in **transverse orientation** over the midportion of the gland with shear wave elastography identifying a mean velocity of 2.44 meters per second (M/sec) (**Figure S4 A, B**). Compression of the gland with firm pressure diminished the depth measurement to 9.0 mm with an increase in the mean shear wave velocity to 3.60 M/sec (**Figure S4 C, D**).

**Figure S4:** Image sequence addressing ultrasound of the left submandibular gland identifies impact of delivered pressure by the ultrasound probe diminishing the width and increasing the density as determined by shear wave elastography (with approval from Hoffman HT (ed) Iowa Head and Neck Protocols < Shear Wave Elastography - Quantitative Salivary Gland Ultrasonography https://medicine.uiowa.edu/iowaprotocols/shear-wave-elastography-quantitative-salivary-glandultrasonography accessed June 18 2023)


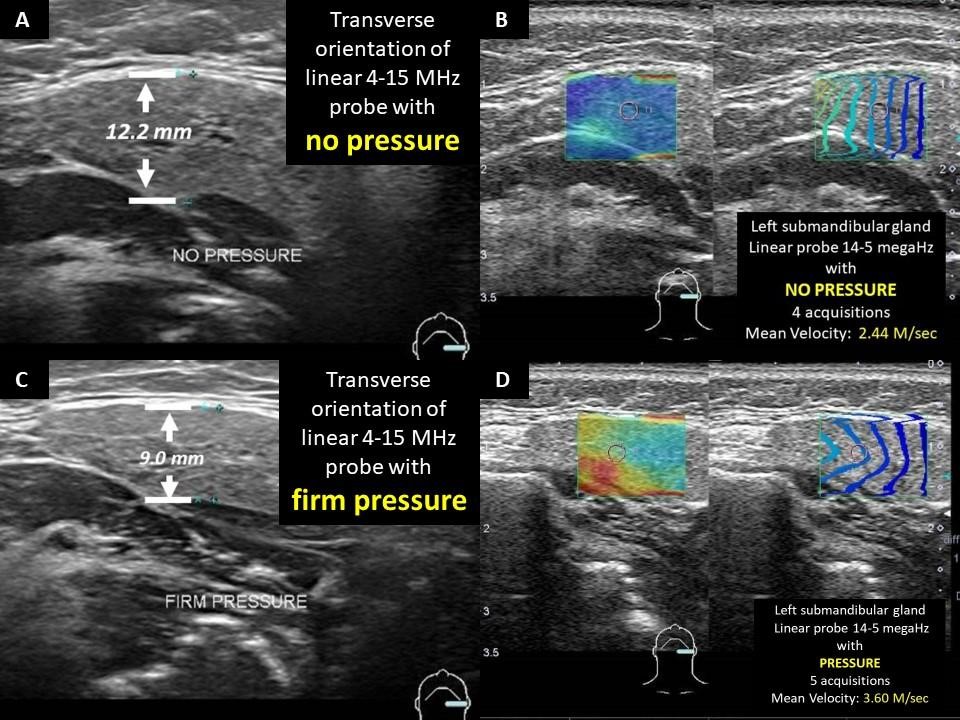


## Cases #2 Indistinct borders to submandibular gland

The finding of the loss of definition of borders has been identified as an impediment to salivary gland size measurement by ultrasound. Recording of this finding in the ultrasound report is important both as a clinically relevant observation and to identify a potential difficulty in assessing the gland size.

### Obesity/Diabetes

As per Badarinza, the normal salivary gland has a regular contour as well as a homogeneous echotexture with intermediate echogenicity.^13^ Through their study comparing healthy patients to a group with diabetes and/or obesity - they found that those in the diabetic and/or obesity group showed increased echogenicity (hyperechoic relative to normal controls), homogeneity (uniform echotexture) and **invisible posterior border** (all p<0.001) **(Figure S5A,B).** All 18 patients in the study with enlarged parotid glands (representing a subgroup with the diagnosis of sialosis) demonstrated moderate to highly increased echogenicity and an invisible posterior border (but no difference in elastography).

These investigators additionally identified: "... *the* ***glandular area of salivary glands in patients with invisible posterior border was sometimes difficult to be assessed*** *(and this can be a source of biases) and the comparison with other imaging techniques would have been useful.*"

**Figure S5A, B** The loss of definition of the deep border in an obese patient (**A**) with increased distance from skin to gland is contrasted to the visible deep border (**B**) in patient with normal BMI. Note the correlation of distance from skin to superficial surface of gland with (13 mm) and without (3.5 mm) the diagnosis of obesity. (with approval adapted from Hoffman HT (ed) Iowa Head and Neck Protocols <

Submandibular Ultrasound - Altered Definition of Gland Margins Due to Obesity, Radiation, Autoimmune Disorder (Sjogren’s) [https://medicine.uiowa.edu/iowaprotocols/submandibularhttps://medicine.uiowa.edu/iowaprotocols/submandibular-ultrasound-altered-definition-gland-margins-due-obesity-radiation-autoimmune-disorderultrasound-altered-definition-gland-margins-due-obesity-radiation-autoimmune-disorder)](https://medicine.uiowa.edu/iowaprotocols/submandibular-ultrasound-altered-definition-gland-margins-due-obesity-radiation-autoimmune-disorder) accessed May 28 2023>.


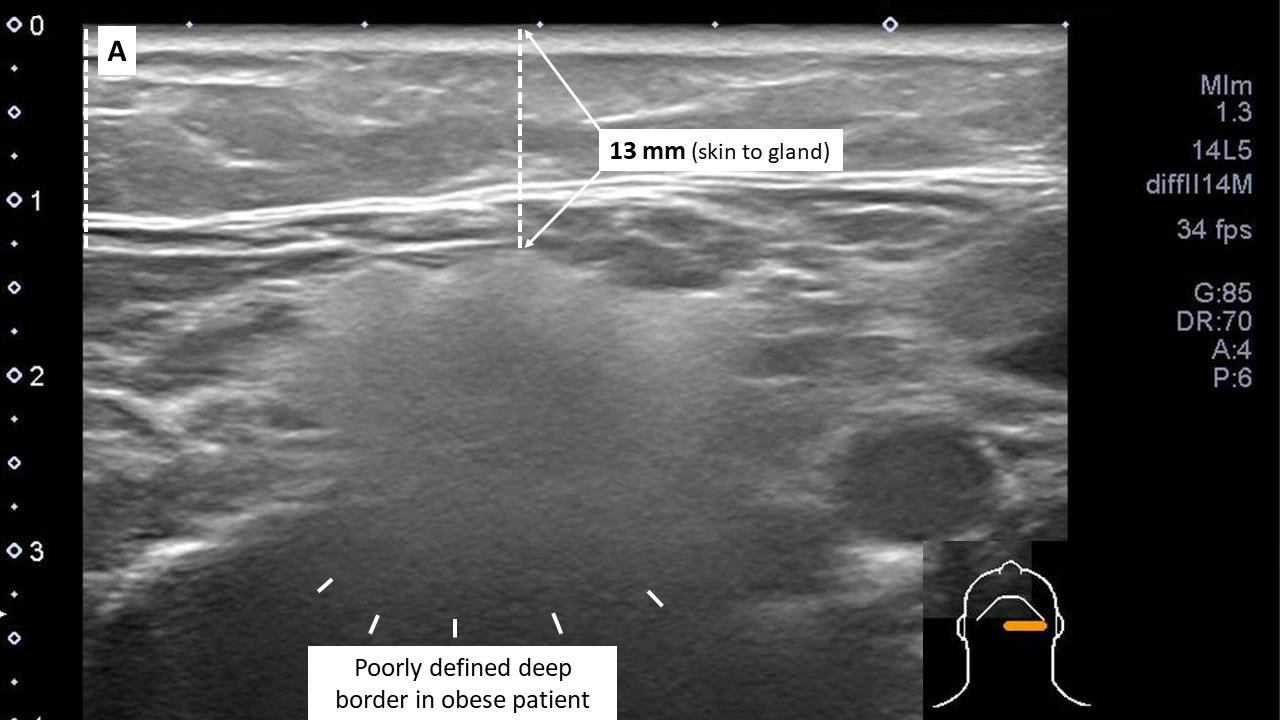


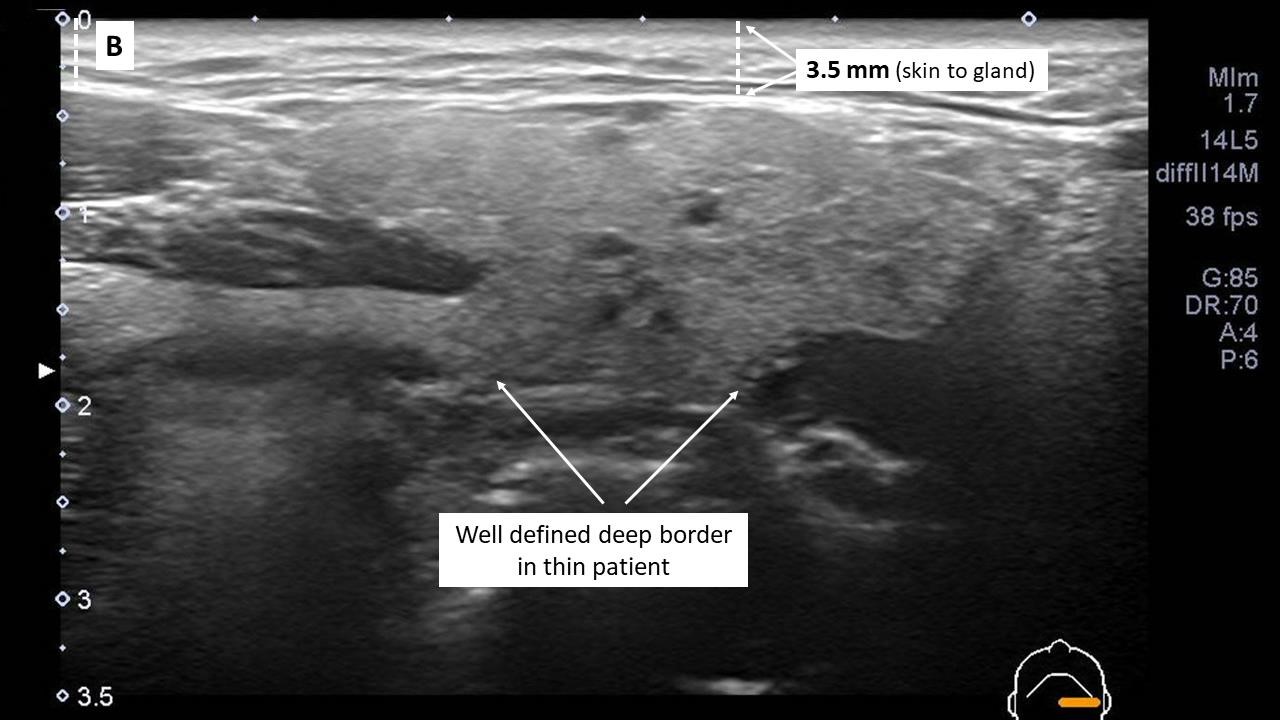


### Irradiation

1. **External beam**

Through ultrasound comparison between patients treated with irradiation for nasopharyngeal cancer (NPC) with normal controls, Cheng et al identified that the majority of the post-RT NPC patients had ill-defined borders to the submandibular glands (89%) in a statistically significant (P<0.05) greater incidence than normal controls.^85^ The investigators postulate that high dose irradiation could destroy the normal capsule to the submandibular gland **(Figure S6)**.

Submandibular gland ultrasound findings reported in association with external beam irradiation following treatment of NPC have included atrophy and abnormal echotexture showing heterogeneity with fatty infiltration.^86^

**Figure S6** Right submandibular gland fibrosis (radiation effect) identified through ultrasound characteristics of a heterogeneous and hypoechoic gland with an irregular poorly defined border. (with approval from Hoffman HT (ed) Iowa Head and Neck Protocols < Submandibular Ultrasound - Altered Definition of Gland Margins Due to Obesity, Radiation, Autoimmune Disorder (Sjogren’s) [https://medicine.uiowa.edu/iowaprotocols/submandibular-ultrasound-altered-definition-glandhttps://medicine.uiowa.edu/iowaprotocols/submandibular-ultrasound-altered-definition-gland-margins-due-obesity-radiation-autoimmune-disordermargins-due-obesity-radiation-autoimmune-disorder)](https://medicine.uiowa.edu/iowaprotocols/submandibular-ultrasound-altered-definition-gland-margins-due-obesity-radiation-autoimmune-disorder) accessed May 28 2023>).


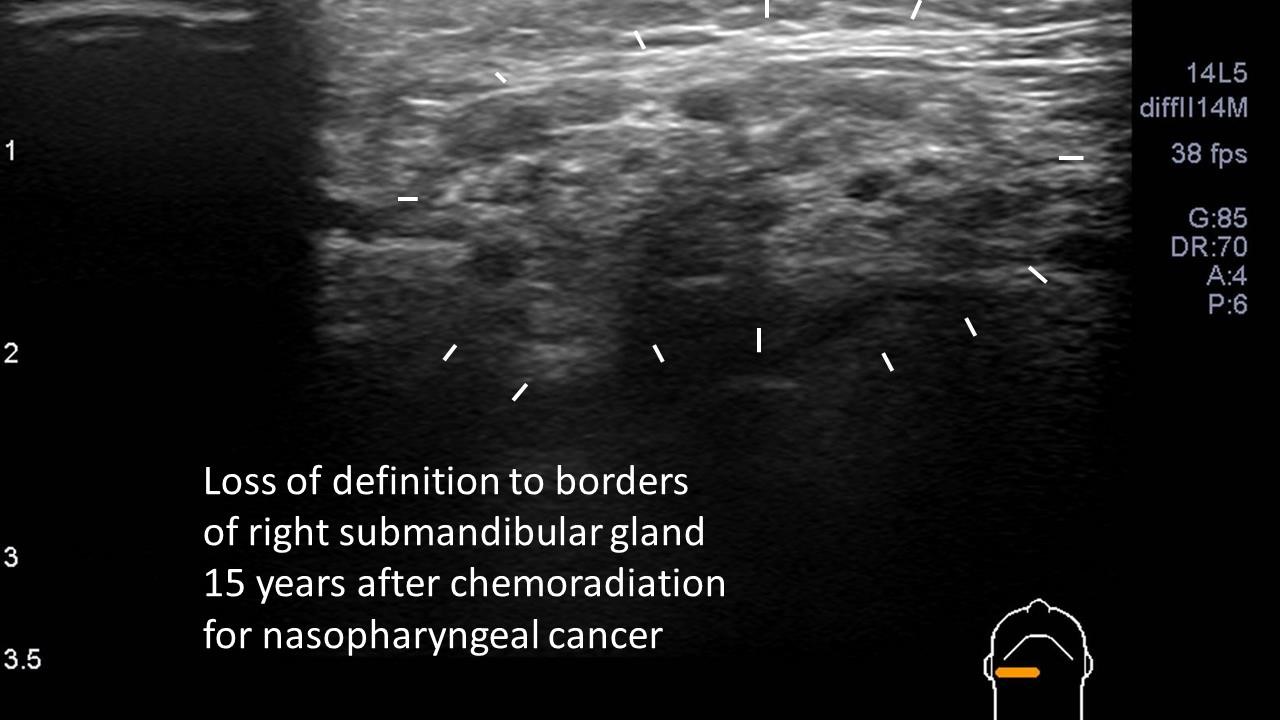


1. **Radioiodine (I131)**

Additionally, radioiodine treatment may be associated with loss of definition to the submandibular gland borders. The clearly defined borders in normal glands may be replaced with an irregular or ‘lobulated’ contour to the submandibular gland – as has been recognized following both external beam radiation and following treatment with radioiodine.^75,85,87-89^ These border irregularities coupled with atrophy and decreased echogenicity may create difficulty in distinguishing the gland from adjacent structures to provide accurate assessment of size.

## Chronic autoimmune sialadenitis (Sjogren's)

Impaired definition of the borders about both the parotid and submandibular glands is also a feature of chronic autoimmune sialadenitis (**Figure S7 A, B)**. The finding of ‘indistinct borders’ has been included in semiquantitative grading systems for ultrasound salivary gland assessment to support the diagnosis of

Sjogren's syndrome.^28,66^ Both contour irregularity and clarity of the posterior (deep) glandular border have been used in multiple scoring systems to contribute to a subjectively defined numerical score to identify abnormalities.^31^ Although the feature of ill-defined or indistinct borders remains a recognized finding, the more recent OMERACT criteria for ultrasound analysis of Sjogren’s syndrome has been truncated to a 4-point system to focus on parenchymal abnormalities and not include border assessment.^90^

**Figure S7 A B** – Example of two patients with Sjogren's syndrome identifying left submandibular gland ultrasound imaging of heterogeneous glands with irregular deep borders. (with approval from Hoffman

HT (ed) Iowa Head and Neck Protocols < Submandibular Ultrasound - Altered Definition of Gland Margins Due to Obesity, Radiation, Autoimmune Disorder (Sjogren’s)

https://medicine.uiowa.edu/iowaprotocols/submandibular-ultrasound-altered-definition-glandmargins-due-obesity-radiation-autoimmune-disorder) accessed May 28 2023>.


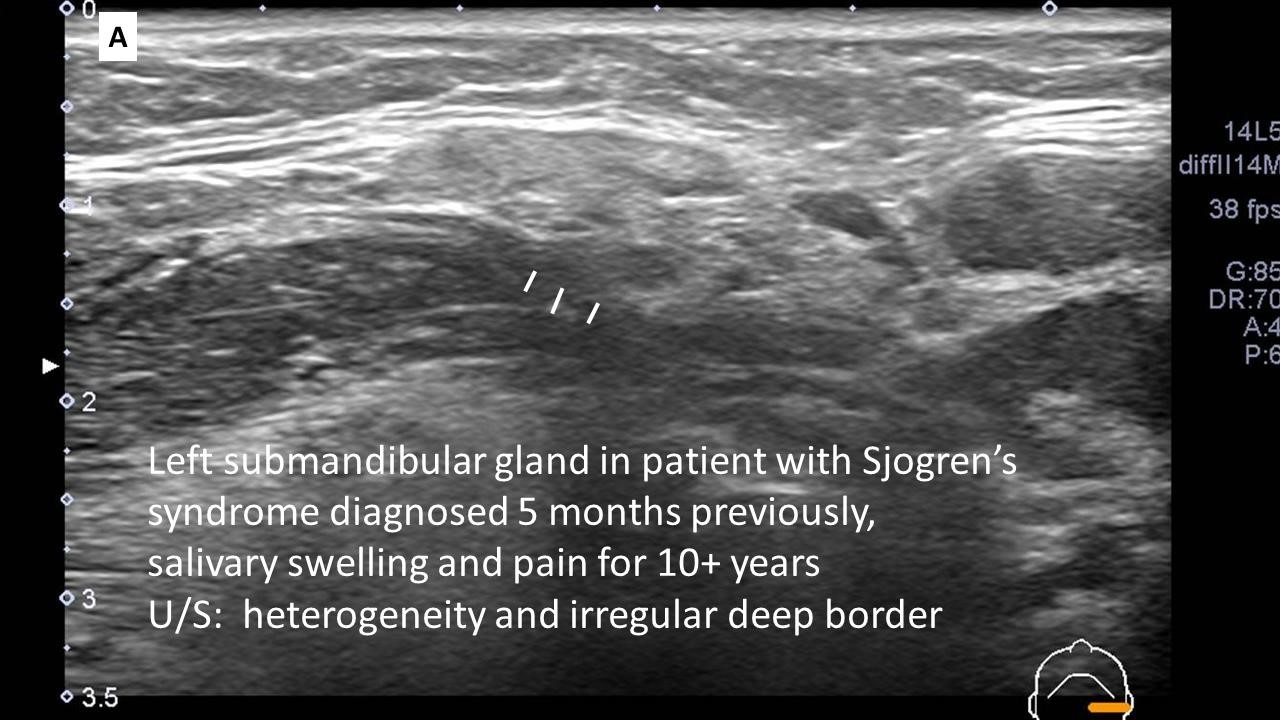


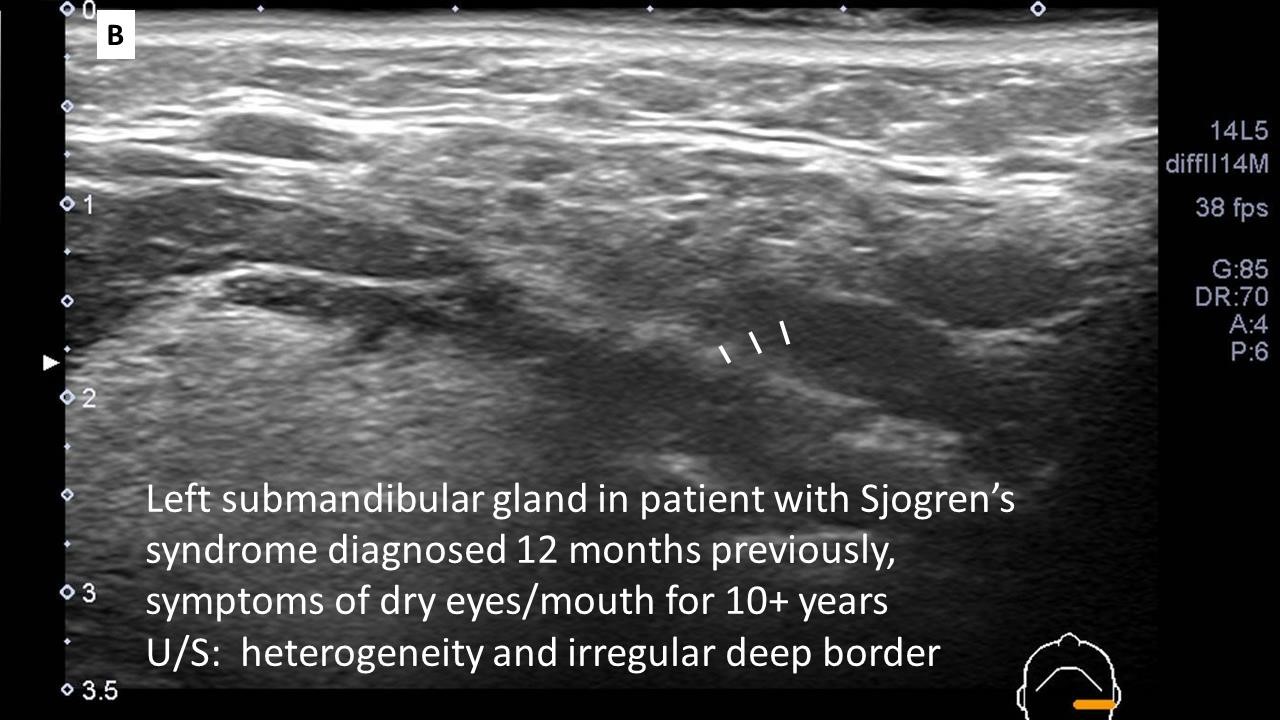


### Subsite identification within the gland

Interpretation of the ultrasound evaluation of the three-dimensional anatomy of the submandibular gland requires additional mental processing to recognize that the superior (cranial) portion of the gland is depicted on the left side of the projected ultrasound image when assessed by longitudinal imaging. Consistency in naming subsites within the gland should help overcome some of the difficulties in interpreting findings in acknowledging that the transverse orientation that is more limited to define the superior border than the longitudinal orientation.

The ***superficial lobe*** of the left submandibular gland was hypoechoic on examination with transvers orientation of the ultrasound probe (**Figure S8**). Fine needle aspiration showed ‘atypical cells’ warranting gland resection showing chronic sialadenitis. The ***deep lobe*** – closest to the ductal drainage system – shows normal hyperechoic and homogeneous echogenicity. The ***superficial lobe in its anterior aspect*** is hypoechoic and heterogeneous with histopathology correlates to these findings.

**Figure S8** Resected submandibular gland correlates with preoperative ultrasound assessment and histopathology. (with approval from Hoffman HT (ed) Iowa Head and Neck

Protocols<https://medicine.uiowa.edu/iowaprotocols/chronic-sialadenitis-histopath-correlateultrasound> accessed June 2, 2023


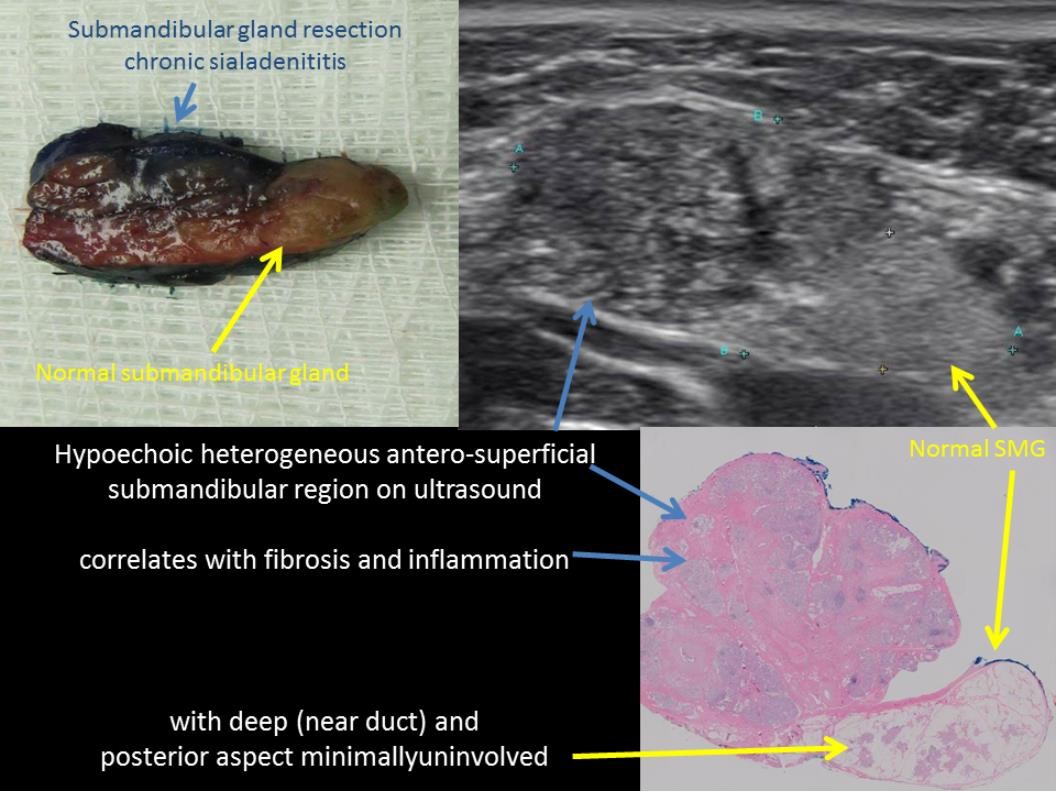


# Discussion

Point-of-care ultrasound (POCUS) analysis was described by Moore and Copel in 2011 as an evaluation performed and interpreted immediately at the bedside by the clinician.^91^ Advances in technology have made ultrasound ‘*user-friendly for all practitioner*s’ with a recent report addressing POCUS by Liao et al supporting it as ‘*essential for clinical practice as well as for training in the field of otolaryngology and head and neck surgery*’.^92^

This AAO-HNS Salivary Gland Committee sponsored proposal aims to standardize technique and nomenclature regarding salivary gland POCUS as practiced in Otolaryngology. However, the recommendations are designed to be broadly applicable to clarify established protocols in other settings including the consultative comprehensive technician-performed examinations done in Radiology departments. Multidisciplinary specialists were called on to refine these recommendations to refine these recommendations to cross boundaries between specialties and support broad acceptance.

This work identifies assessment techniques and define subsites of the submandibular gland in anticipation that this process will improve consistency in performing and reporting ultrasound evaluations. This approach to standardization focuses on static images but is also applicable to terminology used in review of videoclips as has been supported by the OMERACT (Outcome Measures for Rheumatoid Arthritis Clinical Trials) an international rheumatology group. OMERACT has published a semiquantitative approach to provide a more global assessment of salivary gland (primarily parotid) abnormalities associated with Sjogren’s disease through review of video-imaging characterize the degree of pathology seen.^25,26,93^ An updated analysis by Tang et al supported the value of applying this OMERACT scoring system to analysis of static images of the salivary glands.^94^ However, their report failed to identify which subsites were analyzed in their review of ‘*typical static grayscale images*’ in their report that broadly described analysis of transverse and longitudinal sections of the parotid glands and longitudinal only for the submandibular glands.

Shear wave elastography in the assessment of liver fibrosis has revolutionized the approach to cirrhosis to markedly diminish the need for liver biopsy.^95^ Similar application of elastography to salivary gland assessment warrants identification of specific sites within the gland to acknowledge that the gland is often affected in a non-uniform fashion. Our work to standardize the naming of sites within the submandibular gland will be augmented through an upcoming process addressing the parotid gland and is anticipated to lead to a more standardized protocol for performing and reporting shear wave elastography analysis as directed to specific portions of the glands.

Consensus regarding the naming of subsites within the submandibular gland is needed to improve communication about abnormalities detected and to direct the use of more sophisticated assessment schemes such as shear wave analysis targeted to specific regions. Consistency in the nomenclature used to identify locations within these subsites should lead to improved reproducibility of findings.

## Conclusion

# The AAO-HNS Salivary Gland Committee in collaboration with international experts proposes a standardized approach to submandibular gland ultrasound analysis including technique and nomenclature.

**References (in EndNote format)**

1. Davis RA, Anson BJ, Budinger JM, Kurth LR. Surgical anatomy of the facial nerve and parotid gland based upon a study of 350 cervicofacial halves. *Surg Gynecol Obstet.* 1956;102(4):385-412.

2. Brouwer CL, Steenbakkers RJ, Bourhis J, et al. CT-based delineation of organs at risk in the head and neck region: DAHANCA, EORTC, GORTEC, HKNPCSG, NCIC CTG, NCRI, NRG Oncology and TROG consensus guidelines. *Radiother Oncol.* 2015;117(1):83-90.

3. Paczona VR, Capala ME, Deák-Karancsi B, et al. Magnetic Resonance Imaging-Based Delineation of Organs at Risk in the Head and Neck Region. *Adv Radiat Oncol.* 2023;8(2):101042.

4. Afzelius P, Nielsen MY, Ewertsen C, Bloch KP. Imaging of the major salivary glands. *Clin Physiol Funct Imaging.* 2016;36(1):1-10.

5. Quer M, Guntinas-Lichius O, Marchal F, et al. Classification of parotidectomies: a proposal of the European Salivary Gland Society. *Eur Arch Otorhinolaryngol.* 2016;273(10):3307-3312.

6. Pujol-Olmo A, Mirapeix RM, Sañudo-Tejero JR, Quer-Agustí M. Description and relationships of the parotid gland levels proposed by the European Salivary Gland Society staging system: an anatomical study. *Surg Radiol Anat.* 2020;42(9):1101-1107.

7. Abdalla-Aslan R, Keshet N, Zadik Y, Aframian DJ, Nadler C. Standardization of terminology, imaging features, and interpretation of CBCT sialography of major salivary glands: a clinical review. *Quintessence Int.* 2021;52(8):728-740.

8. Lorenzon M, Spina E, Tulipano Di Franco F, Giovannini I, De Vita S, Zabotti A. Salivary Gland Ultrasound in Primary Sjögren's Syndrome: Current and Future Perspectives. *Open Access Rheumatol.* 2022;14:147-160.

9. Biometry. In. *Merriam-Webster*2023.

10. Sheikh K, Lee SH, Cheng Z, et al. Predicting acute radiation induced xerostomia in head and neck Cancer using MR and CT Radiomics of parotid and submandibular glands. *Radiat Oncol.* 2019;14(1):131.

11. Sim C, Soong YL, Pang E, et al. Xerostomia, salivary characteristics and gland volumes following intensity-modulated radiotherapy for nasopharyngeal carcinoma: a two-year follow up. *Aust Dent J.* 2018;63(2):217-223.

12. Patel NJ, Hashemi S, Joshi AS. Sonopalpation: a novel application of ultrasound for detection of submandibular calculi. *Otolaryngol Head Neck Surg.* 2014;151(5):770-775.

13. Badarinza M, Serban O, Maghear L, et al. Multimodal ultrasound investigation (grey scale, Doppler and 2D-SWE) of salivary and lacrimal glands in healthy people and patients with diabetes mellitus and/or obesity, with or without sialosis. *Med Ultrason.* 2019;21(3):257-264.

14. Wiemker R, Rogalla P, Blaffert T, et al. Aspects of computer-aided detection (CAD) and volumetry of pulmonary nodules using multislice CT. *Br J Radiol.* 2005;78 Spec No 1:S46-56.

15. Bozzato A, Burger P, Zenk J, Uter W, Iro H. Salivary gland biometry in female patients with eating disorders. *Eur Arch Otorhinolaryngol.* 2008;265(9):1095-1102.

16. Cardona I, Saint-Martin C, Daniel SJ. Salivary glands of healthy children versus sialorrhea children, is there an anatomical difference? An ultrasonographic biometry. *Int J Pediatr Otorhinolaryngol.* 2015;79(5):644-647.

17. Manetti L, Bogazzi F, Brogioni S, et al. Submandibular salivary gland volume is increased in patients with acromegaly. *Clin Endocrinol (Oxf).* 2002;57(1):97-100.

18. Dost P, Kaiser S. Ultrasonographic biometry in salivary glands. *Ultrasound Med Biol.* 1997;23(9):1299-1303.

19. Açıkgöz G, Akgül HM, Kızıltunç Özmen H, Sezen O. Assessment of Dimensional Changes in Submandibular Glands in Head-Neck Radiotherapy Patients by Ultrasonography. *J Ultrasound Med.* 2023;42(5):1065-1073.

20. Fang KM, Wen MH, Hsu WL, Chang CM, Hou PY, Liao LJ. Ultrasonographic and elastographic biometry in adult major salivary glands: a preliminary case-control report. *Sci Rep.* 2019;9(1):8885.

21. contributors W. Radiomics. *Wikipedia, The Free Encyclopedia*. <https://en.wikipedia.org/w/index.php?title=Radiomics&oldid=1172675394>. Published 2023. Accessed December 25, 2022.

22. Nardone V, Tini P, Nioche C, et al. Texture analysis as a predictor of radiation-induced xerostomia in head and neck patients undergoing IMRT. *Radiol Med.* 2018;123(6):415-423.

23. van Dijk LV, Brouwer CL, van der Schaaf A, et al. CT image biomarkers to improve patient-specific prediction of radiation-induced xerostomia and sticky saliva. *Radiother Oncol.* 2017;122(2):185-191.

24. Jia Y, Yang J, Zhu Y, et al. Ultrasound-based radiomics: current status, challenges and future opportunities. *Med Ultrason.* 2022;24(4):451-460.

25. Hočevar A, Bruyn GA, Terslev L, et al. Development of a new ultrasound scoring system to evaluate glandular inflammation in Sjögren's syndrome: an OMERACT reliability exercise. *Rheumatology (Oxford).* 2022;61(8):3341-3350.

26. Jousse-Joulin S, D'Agostino MA, Nicolas C, et al. Video clip assessment of a salivary gland ultrasound scoring system in Sjögren's syndrome using consensual definitions: an OMERACT ultrasound working group reliability exercise. *Ann Rheum Dis.* 2019;78(7):967-973.

27. Salaffi F, Argalia G, Carotti M, Giannini FB, Palombi C. Salivary gland ultrasonography in the evaluation of primary Sjögren's syndrome. Comparison with minor salivary gland biopsy. *J Rheumatol.* 2000;27(5):1229-1236.

28. Milic VD, Petrovic RR, Boricic IV, et al. Major salivary gland sonography in Sjögren's syndrome: diagnostic value of a novel ultrasonography score (0-12) for parenchymal inhomogeneity. *Scand J Rheumatol.* 2010;39(2):160-166.

29. Zhang X, Zhang S, He J, et al. Ultrasonographic evaluation of major salivary glands in primary Sjögren's syndrome: comparison of two scoring systems. *Rheumatology (Oxford).* 2015;54(9):1680-1687.

30. Elbeblawy YM, Eshaq Amer Mohamed M. Strain and shear wave ultrasound elastography in evaluation of chronic inflammatory disorders of major salivary glands. *Dentomaxillofac Radiol.* 2020;49(3):20190225.

31. Martel A, Coiffier G, Bleuzen A, et al. What is the best salivary gland ultrasonography scoring methods for the diagnosis of primary or secondary Sjögren's syndromes? *Joint Bone Spine.* 2019;86(2):211-217.

32. <standard presentation and labelign of ultrasound images.pdf>.

33. Hofauer B, Roth A, Heiser C, et al. Point Shear Wave Elastography in Diagnosis and Follow-Up of Salivary Gland Affection after Head and Neck Cancer Treatment. *J Clin Med.* 2022;11(21).

34. Knopf A, Hofauer B, Thürmel K, et al. Diagnostic utility of Acoustic Radiation Force Impulse (ARFI) imaging in primary Sjoegren`s syndrome. *Eur Radiol.* 2015;25(10):3027-3034.

35. Bamber J, Cosgrove D, Dietrich CF, et al. EFSUMB guidelines and recommendations on the clinical use of ultrasound elastography. Part 1: Basic principles and technology. *Ultraschall Med.* 2013;34(2):169-184.

36. Shiina T, Nightingale KR, Palmeri ML, et al. WFUMB guidelines and recommendations for clinical use of ultrasound elastography: Part 1: basic principles and terminology. *Ultrasound Med Biol.* 2015;41(5):1126-1147.

37. Hetzel G, Lang W, Strobel D. Atlas of Head and Neck Ultrasound. In: Iro H, Bozzato A, Zenk J, eds. Stuttgart: Georg Thieme Verlag KG; 2013: <http://www.thieme-connect.de/products/ebooks/book/10.1055/b-002-91659>.

38. Jousse-Joulin S, Devauchelle-Pensec V, Cornec D, et al. Brief Report: Ultrasonographic Assessment of Salivary Gland Response to Rituximab in Primary Sjögren's Syndrome. *Arthritis Rheumatol.* 2015;67(6):1623-1628.

39. Blitzer GC, Rogus-Pulia NM, Mattison RJ, et al. Marrow-Derived Autologous Stromal Cells for the Restoration of Salivary Hypofunction (MARSH): Study protocol for a phase 1 dose-escalation trial of patients with xerostomia after radiation therapy for head and neck cancer: MARSH: Marrow-Derived Autologous Stromal Cells for the Restoration of Salivary Hypofunction. *Cytotherapy.* 2022;24(5):534-543.

40. Lynggaard CD, Grønhøj C, Christensen R, et al. Intraglandular Off-the-Shelf Allogeneic Mesenchymal Stem Cell Treatment in Patients with Radiation-Induced Xerostomia: A Safety Study (MESRIX-II). *Stem Cells Transl Med.* 2022;11(5):478-489.

41. Mohamed NH, Shawkat S, Moussa MS, Ahmed N. Regeneration potential of bone marrow derived mesenchymal stem cells and platelet rich plasma (PRP) on irradiation-induced damage of submandibular salivary gland in albino rats. *Tissue Cell.* 2022;76:101780.

42. Sun T, Liu S, Yang G, et al. Mesenchymal stem cell transplantation alleviates Sjögren's syndrome symptoms by modulating Tim-3 expression. *Int Immunopharmacol.* 2022;111:109152.

43. Wang SL, Gao RT. Gene transfer-mediated functional restoration for irradiated salivary glands. *Chin J Dent Res.* 2011;14(1):7-13.

44. Wang Z, Zourelias L, Wu C, Edwards PC, Trombetta M, Passineau MJ. Ultrasound-assisted nonviral gene transfer of AQP1 to the irradiated minipig parotid gland restores fluid secretion. *Gene Ther.* 2015;22(9):739-749.

45. Baum BJ, Zheng C, Cotrim AP, et al. Transfer of the AQP1 cDNA for the correction of radiation-induced salivary hypofunction. *Biochim Biophys Acta.* 2006;1758(8):1071-1077.

46. Bamba R, Shadfar S, Van Natta BW. Fat Grafting as a Novel Treatment for Xerostomia. *J Craniofac Surg.* 2021;32(2):e211-e215.

47. Seror R, Ravaud P, Bowman SJ, et al. EULAR Sjogren's syndrome disease activity index: development of a consensus systemic disease activity index for primary Sjogren's syndrome. *Ann Rheum Dis.* 2010;69(6):1103-1109.

48. Möller I, Janta I, Backhaus M, et al. The 2017 EULAR standardised procedures for ultrasound imaging in rheumatology. *Ann Rheum Dis.* 2017;76(12):1974-1979.

49. Dawes C. Circadian rhythms in human salivary flow rate and composition. *J Physiol.* 1972;220(3):529-545.

50. Papagerakis S, Zheng L, Schnell S, et al. The circadian clock in oral health and diseases. *J Dent Res.* 2014;93(1):27-35.

51. Waterhouse JP, Chisholm DM, Winter RB, Patel M, Yale RS. Replacement of functional parenchymal cells by fat and connective tissue in human submandibular salivary glands: an age-related change. *J Oral Pathol.* 1973;2(1):16-27.

52. Merlo C, Bohl L, Carda C, Gómez de Ferraris ME, Carranza M. Parotid sialosis: morphometrical analysis of the glandular parenchyme and stroma among diabetic and alcoholic patients. *J Oral Pathol Med.* 2010;39(1):10-15.

53. Gray H. *Anatomy of the human body / by Henry Gray.* 30th American edition ed. Philadelphia: Philadelphia : Lea & Febiger; 1985.

54. Hollinshead WH. *Anatomy for Surgeons.* Vol 1 The Head and Neck. 2nd ed. New York: New York : Hoeber Medical Division, Harper & Row; 1968.

55. Xu MJ, Chang JL. Practical Salivary Ultrasound Imaging Tips and Pearls. *Otolaryngol Clin North Am.* 2021;54(3):471-487.

56. Ogle OE. Excision of Sublingual Gland. *Oral Maxillofac Surg Clin North Am.* 2021;33(2):161-168.

57. Yves Saban TS, Peter Palhazi, Roberto Polselli. *Surgery of the Salivary Glands Chapter 1: Salivary Gland Anatomy.* Edinburgh: Elsevier; 2021.

58. Atkinson C, Fuller J, 3rd, Huang B. Cross-Sectional Imaging Techniques and Normal Anatomy of the Salivary Glands. *Neuroimaging Clin N Am.* 2018;28(2):137-158.

59. M M, A K. *Contemporary oral and maxillofacial surgery Chapter 21: A Diagnosis and Management of Salivary Gland Disorders.* Seventh edition.. ed. St. Louis: St. Louis : Elsevier; 2018.

60. Ugalde P, Camargo Jde J, Deslauriers J. Lobes, fissures, and bronchopulmonary segments. *Thorac Surg Clin.* 2007;17(4):587-599.

61. Bismuth H. Revisiting liver anatomy and terminology of hepatectomies. *Ann Surg.* 2013;257(3):383-386.

62. Juza RM, Pauli EM. Clinical and surgical anatomy of the liver: a review for clinicians. *Clin Anat.* 2014;27(5):764-769.

63. Garcia-Serrano G, Moñux A, Maranillo E, et al. Vascular clinical anatomy of the submandibular gland. *J Craniomaxillofac Surg.* 2020;48(6):582-589.

64. Yazbeck A, Iwanaga J, Walocha JA, Olewnik Ł, Tubbs RS. The clinical anatomy of the accessory submandibular gland: a comprehensive review. *Anat Cell Biol.* 2023;56(1):9-15.

65. O'Daniel TG. Understanding Deep Neck Anatomy and Its Clinical Relevance. *Clin Plast Surg.* 2018;45(4):447-454.

66. Hocevar A, Ambrozic A, Rozman B, Kveder T, Tomsic M. Ultrasonographic changes of major salivary glands in primary Sjogren's syndrome. Diagnostic value of a novel scoring system. *Rheumatology (Oxford).* 2005;44(6):768-772.

67. Koch M, Sievert M, Iro H, Mantsopoulos K, Schapher M. Ultrasound in Inflammatory and Obstructive Salivary Gland Diseases: Own Experiences and a Review of the Literature. *J Clin Med.* 2021;10(16).

68. Leppi TJ. Gross anatomical relationships between primate submandibular and sublingual salivary glands. *J Dent Res.* 1967;46(2):359-365.

69. Katz P, Hartl DM, Guerre A. Clinical ultrasound of the salivary glands. *Otolaryngol Clin North Am.* 2009;42(6):973-1000, Table of Contents.

70. Li L, Gao XL, Song YZ, et al. Anatomy of arteries and veins of submandibular glands. *Chin Med J (Engl).* 2007;120(13):1179-1182.

71. MacLeod AM, Robbins SP. Submandibular gland transfer in the correction of dry eye. *Aust N Z J Ophthalmol.* 1992;20(2):99-103.

72. Paniello RC. Submandibular gland transfer for severe xerophthalmia. *Laryngoscope.* 2007;117(1):40-44.

73. AIUM-ACR-SPR-SRU Practice Parameter for the Performance and Interpretation of a Diagnostic Ultrasound Examination of the Extracranial Head and Neck. *J Ultrasound Med.* 2018;37(11):E6-e12.

74. Marteau P, Cornec D, Gouillou M, et al. Assessment of major salivary gland size in primary Sjögren's syndrome: Comparison between clinical examination and ultrasonography. *Joint Bone Spine.* 2019;86(5):627-632.

75. Imanimoghaddam M, Rahrooh M, Tafakhori Z, Zahedanaraki S, Homaeieshandiz F. Changes of parotid and submandibular glands caused by radiotherapy--an ultrasound evaluation. *Dentomaxillofac Radiol.* 2012;41(5):379-384.

76. Bahner DP, Blickendorf JM, Bockbrader M, et al. Language of Transducer Manipulation: Codifying Terms for Effective Teaching. *J Ultrasound Med.* 2016;35(1):183-188.

77. Rago T, Chiovato L, Grasso L, Pinchera A, Vitti P. Thyroid ultrasonography as a tool for detecting thyroid autoimmune diseases and predicting thyroid dsfunction in apparently healthy subjects. *J Endocrinol Invest.* 2001;24(10):763-769.

78. Stevenson JH. Tissue Scanning. In: Daniels JM, Dexter WW, eds. *Basics of Musculoskeletal Ultrasound.* New York, NY: Springer New York; 2013:15-27.

79. Blessing M. Ultrasound Probe Selection, Knobology and Optimization of Image Quality. In: Li J, Ming-Der Chow R, Vadivelu N, Kaye AD, eds. *Ultrasound Fundamentals : An Evidence-Based Guide for Medical Practitioners.* Cham: Springer International Publishing; 2021:17-24.

80. Situ-LaCasse E, Acuña J. Principles of Ultrasound Guidance. In: Adhikari S, Blaivas M, eds. *The Ultimate Guide to Point-of-Care Ultrasound-Guided Procedures.* Cham: Springer International Publishing; 2020:5-27.

81. Etter LE. *Glossary of Words and Phrases Used in Radiology, Nuclear Medicine, and Ultrasound: Prepared from Various Sources for Medical Secretaries, X-ray Technicians, Medical Students, and Residents in Radiology.* C.C. Thomas; 1970.

82. Hoffman HT, Pagedar NA. Ultrasound-Guided Salivary Gland Techniques and Interpretations. *Atlas Oral Maxillofac Surg Clin North Am.* 2018;26(2):119-132.

83. Hall MM, Allen GM, Allison S, et al. Recommended Musculoskeletal and Sports Ultrasound Terminology: A Delphi-Based Consensus Statement. *J Ultrasound Med.* 2022;41(10):2395-2412.

84. Goncalves M, Schapher M, Iro H, Wuest W, Mantsopoulos K, Koch M. Value of Sonography in the Diagnosis of Sialolithiasis: Comparison With the Reference Standard of Direct Stone Identification. *J Ultrasound Med.* 2017;36(11):2227-2235.

85. Cheng SC, Ying MT, Kwong DL, Wu VW. Sonographic appearance of submandibular glands in patients treated with external beam radiotherapy for nasopharyngeal carcinoma. *J Clin Ultrasound.* 2013;41(8):472-478.

86. King AD, Ahuja AT, Yeung DK, et al. Delayed complications of radiotherapy treatment for nasopharyngeal carcinoma: imaging findings. *Clin Radiol.* 2007;62(3):195-203.

87. Orloff L, Hwang H, Jecker P. The role of ultrasound in the diagnosis and management of salivary disease. *Operative Techniques in Otolaryngology-Head and Neck Surgery.* 2009;20(2):136-144.

88. Horvath E, Skoknic V, Majlis S, et al. Radioiodine-Induced Salivary Gland Damage Detected by Ultrasonography in Patients Treated for Papillary Thyroid Cancer: Radioactive Iodine Activity and Risk. *Thyroid.* 2020;30(11):1646-1655.

89. Tsur N, Avishai G, Alkan U, et al. Ultrasonographic Features of Salivary Glands after Radioiodine Therapy in Patients with Thyroid Cancer. *Laryngoscope.* 2023;133(5):1271-1275.

90. Finzel S, Jousse-Joulin S, Costantino F, et al. Patient-based reliability of the Outcome Measures in Rheumatology (OMERACT) ultrasound scoring system for salivary gland assessment in patients with Sjögren's syndrome. *Rheumatology (Oxford).* 2021;60(5):2169-2176.

91. Moore CL, Copel JA. Point-of-care ultrasonography. *N Engl J Med.* 2011;364(8):749-757.

92. Liao LJ, Wen MH, Yang TL. Point-of-care ultrasound in otolaryngology and head and neck surgery: A prospective survey study. *J Formos Med Assoc.* 2021;120(8):1547-1553.

93. Cornec D, Jousse-Joulin S, Pers JO, et al. Contribution of salivary gland ultrasonography to the diagnosis of Sjögren's syndrome: toward new diagnostic criteria? *Arthritis Rheum.* 2013;65(1):216-225.

94. Tang G, Luo Y, Mo Y, Yao J, Yang H, Hao S. Diagnostic value of ultrasound evaluation of major salivary glands for Sjögren's syndrome based on the novel OMERACT scoring system. *Eur J Radiol.* 2023;162:110765.

95. Tamaki N, Kurosaki M, Huang DQ, Loomba R. Noninvasive assessment of liver fibrosis and its clinical significance in nonalcoholic fatty liver disease. *Hepatol Res.* 2022;52(6):497-507.
